# The Brazilian Portuguese Lexicon: An Instrument for Psycholinguistic Research

**DOI:** 10.1371/journal.pone.0144016

**Published:** 2015-12-02

**Authors:** Gustavo L. Estivalet, Fanny Meunier

**Affiliations:** 1 CNRS UMR5304, Laboratoire sur le Langage, le Cerveau et la Cognition, Institut de Sciences Cognitives, Bron, France; 2 Université Claude Bernard Lyon 1, Université de Lyon, Lyon, France; University of Groningen, NETHERLANDS

## Abstract

In this article, we present the Brazilian Portuguese Lexicon, a new word-based corpus for psycholinguistic and computational linguistic research in Brazilian Portuguese. We describe the corpus development, the specific characteristics on the internet site and database for user access. We also perform distributional analyses of the corpus and comparisons to other current databases. Our main objective was to provide a large, reliable, and useful word-based corpus with a dynamic, easy-to-use, and intuitive interface with free internet access for word and word-criteria searches. We used the *Núcleo Interinstitucional de Linguística Computacional*’s corpus as the basic data source and developed the Brazilian Portuguese Lexicon by deriving and adding metalinguistic and psycholinguistic information about Brazilian Portuguese words. We obtained a final corpus with more than 30 million word tokens, 215 thousand word types and 25 categories of information about each word. This corpus was made available on the internet via a free-access site with two search engines: a simple search and a complex search. The simple engine basically searches for a list of words, while the complex engine accepts all types of criteria in the corpus categories. The output result presents all entries found in the corpus with the criteria specified in the input search and can be downloaded as a.csv file. We created a module in the results that delivers basic statistics about each search. The Brazilian Portuguese Lexicon also provides a pseudoword engine and specific tools for linguistic and statistical analysis. Therefore, the Brazilian Portuguese Lexicon is a convenient instrument for stimulus search, selection, control, and manipulation in psycholinguistic experiments, as also it is a powerful database for computational linguistics research and language modeling related to lexicon distribution, functioning, and behavior.

## Introduction

Word-based corpora are extremely important in providing metalinguistic, psycholinguistic, and statistical information about lexicons. They are used for selecting, controlling, and manipulating words in psycholinguistic experiments [1–4]. Furthermore, word-based corpora provide rich databases of language behavior and lexicon distribution for computational linguistics analyses [5,6] and language modeling [7,8]. In this article, we present the Brazilian Portuguese Lexicon <http://www.lexicodoportugues.com/>, *Léxico do Português Brasileiro (LexPorBR)* in Portuguese, a new instrument for metalinguistic and psycholinguistic research that provides information and statistics about Brazilian Portuguese words. A number of similar corpora are currently available for many languages, e.g., English, MRC [9] <http://www.psych.rl.ac.uk/>; French, *Lexique* [4] <http://www.lexique.org/>; Spanish, *BuscaPalabras* [10]; Dutch, English, and German, CELEX [3] <http://celex.mpi.nl/>; and Dutch, English, French, German, and Spanish, ClearPOND [2] <http://clearpond.northwestern.edu/>. Further, large databases have been developed for many languages from subtitles, e.g., American English [11], British English [12], Dutch [13], Chinese [14], and Brazilian Portuguese [15]: SUBTLEX <http://crr.ugent.be/programs-data/subtitle-frequencies>; and from internet information, e.g., 66 languages: Worldlex [16]. However, one limitation of some subtitle and internet databases is that they are not Part-Of-Speech (POS) tagged, presenting no grammatical category, grammatical information or homographs, and providing only word frequency. Differently, POS-tagged psycholinguistic word-based corpora like CELEX [3], *Lexique* [4], and SUBTLEX-UK [12], which contain many metalinguistic and psycholinguistic information about words, are very useful and affordable for many purposes, but did not exist for Brazilian Portuguese. Although the *Linguateca* <http://www.linguateca.pt/> contains a large number of Portuguese corpora, most of them are from European Portuguese, none is word-based, and none provides specific information for the purposes described above [17]. Aiming to fill this gap in the descriptive and linguistic data regarding the Brazilian Portuguese language, we constructed the Brazilian Portuguese Lexicon. It provides a great deal of metalinguistic, psycholinguistic, and statistical information [18,19] in a corpus containing more than 30 million word tokens, more than 215 thousand word types, more than 160 thousand lemmas, more than 290 thousand proper names, and 25 columns of information. All of these data, information, and search facilities were delivered in a free, open-access corpus on an internet site.

Portuguese is an inflectional Romance language established in the 12^th^ century on the Iberian Peninsula in the Kingdom of Galicia, being expanded to Africa, Asia, and South America through colonization from the 15^th^ century. It is the third most spoken language in Europe and Western Hemisphere and the first most spoken language in South America and Southern Hemisphere [20]. Brazilian Portuguese (BP) is spoken by more than 200 million native speakers and presents many differences from European Portuguese (EP) (e.g., a) syntax: BP: *Estou escrevendo* ‘I am writing’, EP: *Estou a escrever* ‘I am to writing’; b) orthography: BP: *ação*, *azilo*, EP: *acção*, *asylo* ‘action, azylum’; c) phonology/prosody: BP: *menino* /me’nino/, EP: *menino* /’mnino/ ‘boy’; d) semantics: BP: *açougue* ‘butchery’, EP: *talho* ‘butchery’ [21,22]; e) frequency: word frequencies in Brazil are different from Portugal [17], and f) Brazilian Portuguese is influenced by many American indigenous languages, presenting many current frequent indigenous words, BP: *abacaxi*, *gambá*, *pipoca* ‘pineapple, skunk, popcorn’).

A number of studies interested in different levels of word recognition and production have been conducted in many languages using stimuli chosen according to specific criteria searched in corpora for each language [23]. For example, morphological decomposition in French was researched in function of surface and base frequencies [24], and lexical decision reaction times (RTs) in French were analyzed using a large number of corpus items [25]; they chose their French stimuli from the *Lexique* [4]. Most psycholinguistic research in Dutch [26,27], English [1,28], and German [29,30] has used stimuli from the CELEX [3]. However, for Brazilian Portuguese, Justi and Roazzi (2012) [31] had to construct their own corpus to select stimuli for their experiments on orthographic neighborhood effects [32], and Sicuro Corrêa, Almeida, and Porto (2004) [33] used accumulated newspaper evidence for their experiments on the representation of gender-inflected words. Therefore, we strongly believe that the Brazilian Portuguese Lexicon will be helpful and useful for searching, controlling, manipulating, and selecting stimuli in psycholinguistic experiments [2,34], for computational linguistic analysis [5,35], and for language modeling [7,8,36,37], thus filling a gap in Brazilian Portuguese research, the lexicon, and language description and information.

**Fig 1:**
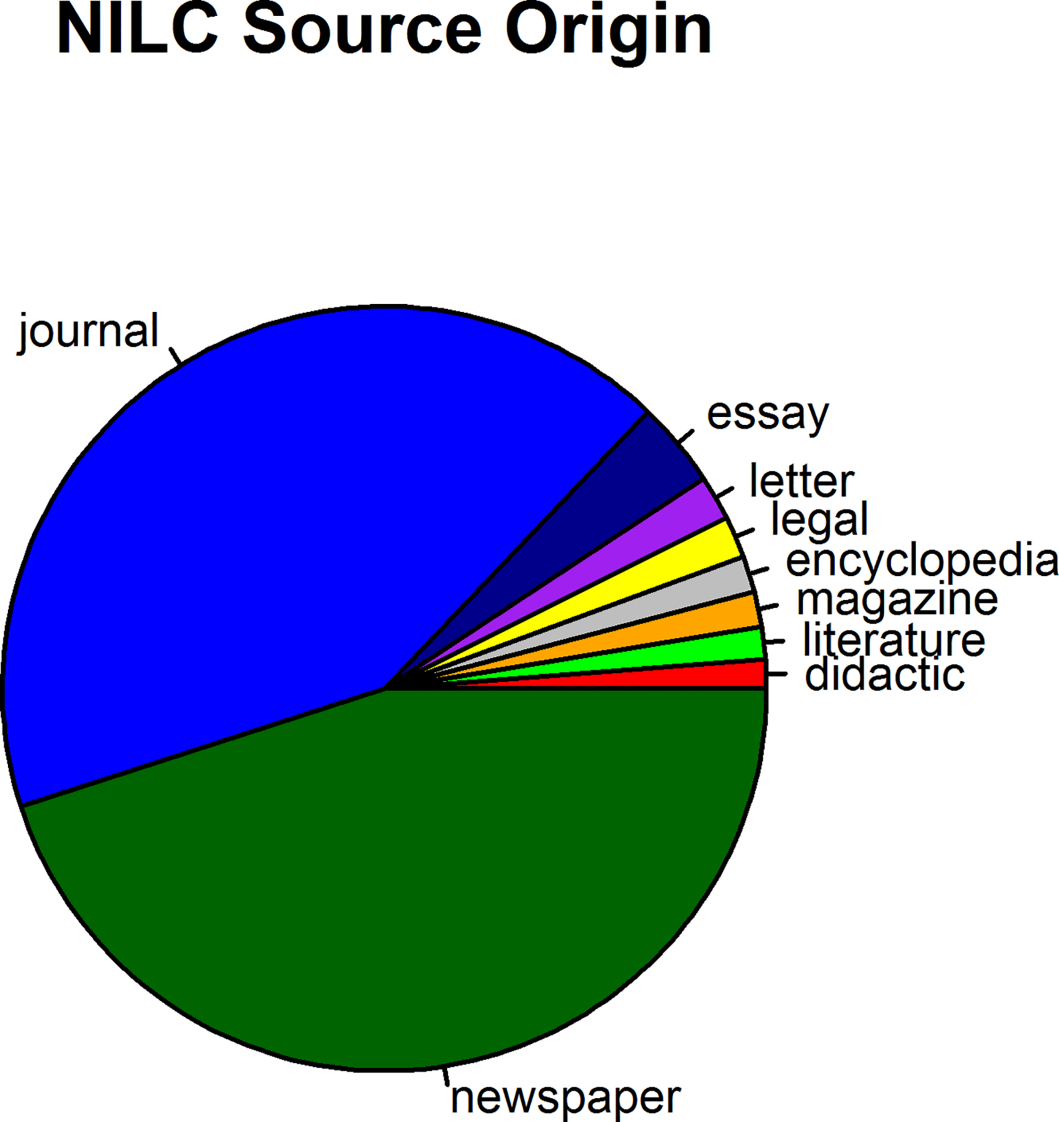
Distribution of the NILC source of origin. Distribution of the different textual genres and written materials that composed the original source of the NILC [38].

We identified the two largest and most representative corpora of Brazilian Portuguese at the *Linguateca*: the *Corpus Brasileiro* <http://corpusbrasileiro.pucsp.br/cb/Inicial.html/> with word tokens of more than 1 billion and the corpus from the *Núcleo Interinstitucional de Linguística Computacional* (NILC), with word tokens of more than 30 million [17]. We decided to use the NILC to develop the Brazilian Portuguese Lexicon based on the following characteristics: a) its total word tokens with more than 30 million [11], in accordance with other psycholinguistic corpus, including CELEX (Dutch, 40 million; English, 18 million; German, 5 million), *Lexique* (30 million), SUBTLEX-CH (47 million), SUBTLEX-NL (44 million), Wolrdlex (Portuguese Brazil, 50 million), and *BuscaPalabras* (5 million), b) its total word types with more than 215 thousand word types, more than 160 thousand lemmas, and more than 290 thousand proper names, c) its file size of 49 MB, d) its processing demands, e) its POS-tagged information about grammatical categories, and f) its source origins <http://www.linguateca.pt/acesso/NILCsaocarlos.html/>, mainly newspapers (45.02%) and journals (42.07%) but also essays (3.65%), letters (1.87%), legal documents (1.77%), encyclopedias (1.54%), magazines (1.48%), literature (1.38%), and didactic (1.22%) sources <http://www.linguateca.pt/acesso/desc_corpus.php?corpus=SAOCARLOS/> as shown in **Fig 1**. The NILC’s source origins are a remarkable collection of written text consisting primarily of the *Folha de São Paulo*’s newspaper database, many other journalistic sources, and a variety of different textual genres [38]. Regarding its size, Brysbaert and New (2009, p.980) [11] stated that “[…] for most practical purposes, a corpus of 16–30 million words suffices for reliable word frequency norms. In particular, there is no evidence that a corpus of 3 billion words is much better than a corpus of 30 million words”; the same finding was robustly replicated in the SUBTLEX-NL [13] and SUBTLEX-UK [12].

The Brazilian Portuguese Lexicon was developed by processing the data from the NILC available in the *Linguateca* using R software version 3.1.2 (R Foundation for Statistical Computing, Vienna, Austria) [39] <http://www.r-project.org/>, ‘vwr’ [40] <http://cran.r-project.org/web/packages/vwr/index.html/>, ‘languageR’ [41] <http://cran.r-project.org/web/packages/languageR/index.html/>, and ‘psych’ [42] <http://cran.r-project.org/web/packages/psych/index.html/> R packages. After establishing the corpus, we imported it to a MySQL database on a free internet server <http://www.biz.nf/> and constructed the Brazilian Portuguese Lexicon internet site using the programming languages HTML, Java, and CSS for the visual interface; and MySQL and PHP for the logical functions and interface between the user and the corpus.

In the next sections, in materials and methods, we explain the data acquisition, organization, and computation, as well as the lexicon construction, and the lexicon information. Afterwards, we describe the internet site, the search engines, the search results, the site resources, and the Creative Commons License. In the results section, we present the general framework of the Brazilian Portuguese Lexicon, the lexicon distributions, and comparisons to other corpora. Then, in the discussion section, we discuss these results in detail, and end with final remarks, limitations, and further developments of the Brazilian Portuguese Lexicon.

## Materials and Methods

### Data acquisition, organization, and computation

Our work was based on 13.txt files (i.e., adjectives, adverbs, grammatical, nouns, numerals, and verbs for forms and lemmas, plus one lemma file with proper names) from the NILC available on the *Linguateca* <http://www.linguateca.pt/acesso/contabilizacao.php#saocarlos/>. The files provide lists of words and their frequencies, as summarized in the first three columns of **Table 1**, giving us a rough idea of the corpus distribution [17,38]. We then concatenated the files, homogenized the entries in lower cases, and checked for repetitions. We removed all numeric entries except for the digits 0–9 and 1^st^–9^th^ as adjectives, nouns, and numerals. We also removed all entries longer than 30 characters, which held no interest for psycholinguistic research and for the corpus. Thus, the corpus initially had a total of 31,377,670 word tokens, 316,101 word types, 169,965 lemmas, and 301,860 proper names. After our processing for the Brazilian Portuguese Lexicon, we were left with a total of 30,705,945 word tokens, 215,175 word types, 160,753 lemmas, and 293,198 proper names, as summarized in the last three columns of **Table 1**. Therefore, we will further consider these values for word tokens, word types, lemmas, and proper names.

**Table 1:** Numbers of word tokens, word types, and lemmas by grammatical category before and after data processing for the Brazilian Portuguese Lexicon.

Gram. Cat.	Tokens NILC	Types^a^ NILC	Lemmas^b^ NILC	Tokens LexPorBR	Types LexPorBR	Lemmas LexPorBR
Adjectives	1,842,597	46,249	24,478	1,829,473	40,537	24,058
Adverbs	1,455,573	3,611	2,857	1,455,573	2,938	2,723
Grammatical	15,717,557	1,809	480	15,702,419	1,144	455
Nouns	7,113,649	100,328	66,189	7,079,524	82,097	64,421
Numerals	949,766	58,672	61,341	340,428	136	54,942
Verbs	4,298,528	105,432	14,620	4,298,528	88,323	14,154
Proper names	-	-	301,860	-	-	293,198
**Total**	**31,377 670**	**316,101**	**471,825**	**30,705,945**	**215,175**	**453,951**

^a^ <http://www.linguateca.pt/acesso/contabilizacao.php#listaPosSAOCARLOS/>.

^b^ <http://www.linguateca.pt/acesso/contabilizacao.php#listaLemasSAOCARLOS/>.

### Lexicon construction

In this section, we summarize the Brazilian Portuguese Lexicon development. The orthographic form (orthography) and the orthographic frequency (ortho_freq) were initially provided by the NILC files, we then added a column with the grammatical category (gram_cat). With the corpus organized by descending orthographic frequency and by orthography (a-z and 0–9), we added a column with an identification number (id) for each entry that automatically became the word’s position in the lexicon [5]; we also added a column containing a random number (random) between 0 and 1 with eight-digit precision for each word. Next, we calculated the orthographic frequency per million (ortho_freq/M) by dividing the orthographic frequency by the total number of words in the corpus (30,705,945) and multiplying by 1 million; and the common logarithmic function (log10_ortho_freq) based on the orthographic frequency. We also calculated the Zipf scale (zipf_scale) by the common logarithmic function based on the orthographic frequency per million plus 3 [12]; and the Zipf’s rank-frequency distribution (zipf_rank) [5,41]. Next, we added different columns with the number of letters (nb_letters), number of homographs (nb_homogr), and the grammatical categories of the homographs (homogr).

We then added columns with the CVCV structure (cvcv_ortho) (where ‘C’ for consonants and ‘V’ for vowels, in addition to ‘N’ for numbers, ‘A’ for accents, ‘P’ for punctuation, and ‘S’ for symbols; see **S1 Text**), bigrams (bigrams) concatenating letters two by two, and trigrams (trigrams) concatenating letters three by three. To determine the first and last bigrams and trigrams of each word, the hash symbol ‘#’ was concatenated as the word’s start and end bounds [37]. We used the underline symbol ‘_’ to separate different bigrams and trigrams because there are a large number of composed words and clitics separated by a hyphen ‘-’ in Portuguese [43,44]. The number of bigrams in a word is the number of letters plus one and the number of trigrams is the same number of letters. Then, we listed in different files all the bigrams and trigrams present in the corpus and calculated their respective frequencies in function of the number of different word types containing these bigrams and trigrams by grammatical category and position; these lists were used in the pseudoword generation engine and are available in the Downloads page. Further, we calculated the bigram frequency (bigram_freq) and trigram frequency (trigram_freq) based on the sum of the bigrams and trigrams frequencies that compose the words [10,18]. We then added reversed columns for orthography (rev_ortho), CVCV orthographic structure (rev_cvcv_ortho), bigrams (rev_bigrams), and trigrams (rev_trigrams).

Next, we developed an algorithm (**S2 Text**) to calculate the orthographic uniqueness point (pu_ortho), by comparing the left-to-right orthography of each word with the whole corpus to determine the letter position at which each word becomes unique in the corpus. Next, we added a column containing the number of orthographic neighborhoods for each word (ortho_neigh), most commonly known as Coltheart’s N, which is determined by calculating the number of words that can be matched in the corpus with the Hamming distance of 1, i.e., by substituting a single letter in any position within the string [32,45]. We added a column for the Orthographic Levenshtein Distance among the 20 closest words in the corpus (old20), which, unlike the binary Coltheart’s N, incorporates a graded and flexible measure of similarity by deleting, adding, and substituting letters [46,47].

Finally, we added a column with grammatical information (gram_inf) that was completed using all of the available grammatical information about the words (i.e., gender, number, class, mood, tense, and person), specifically identifying and using suffixes that can be categorized accordingly to Portuguese dictionaries, grammars, and morphological manuals [21,22,43,44]. The R algorithm (**S2 Text**) presents the algorithm that was developed in the R software [39], which can be used and adapted for additional applications.

### Lexicon information

A number of columns that contain psycholinguistic and metalinguistic information about each Brazilian Portuguese word in the corpus were computed and added to the database. **Table 2** summarizes all 25 columns that were created and describes the information they contain.

**Table 2:** Numbers, columns, and descriptions of the Brazilian Portuguese Lexicon.

Nb	Column	Description
1	orthography	Orthographic representation
2	gram_cat	Grammatical category
3	gram_inf	Grammatical information
4	ortho_freq	Orthographic frequency
5	ortho_freq/M	Orthographic frequency per million
6	log10_ortho_freq	Log10 from ortho_freq
7	zipf_scale	Standardized frequency scale
8	zipf_rank	Zipf’s rank-frequency distribution
9	nb_letters	Number of letters
10	nb_homogr	Number of homographs
11	homographs	Homograph grammatical categories
12	pu_ortho	Orthographic uniqueness point
13	ortho_neigh	Orthographic neighborhood
14	old20	Orthographic Levenshtein Distance 20 words
15	cvcv_ortho	Consonant/vowel CVCV structure
16	bigrams	Bigrams representation
17	bigram_freq	Bigram frequency
18	trigrams	Trigrams representation
19	trigram_freq	Trigram frequency
20	rev_ortho	Reverse orthography
21	rev_cvcv_ortho	Reverse CVCV structure
22	rev_bigrams	Reverse bigrams
23	rev_trigrams	Reverse trigrams
24	random	Random number between 0–1
25	id	Identity number (position)

Orthography column presents the orthographic form of each word in the corpus (e.g., *cachorro*, ‘dog’). Grammatical category shows the grammatical category to which the word belongs, according to the first seven rows in **Table 3** (e.g., *cachorro* = nom). Grammatical information provides all grammatical information for each entry (i.e., gender, number, class, mood, tense, and person), according to the conventions listed in **Table 3** (e.g., *cachorro* = m, s [masculine, singular]) [21,22,43,44,48].

**Table 3:** Conventions used in the grammatical category and grammatical information columns in the search engines and results at the Brazilian Portuguese Lexicon.

Convention	Meaning	Example
adj	adjective	caro, jovem, linda, velho
adv	adverb	quase, sempre, seguido, também
gram	grammatical	com, depois, para, que
nom	noun	cachorro, dedo, roda, tábua
num	numeral	3, 9, 1°, 8ª
ver	verb	comer, deitou, ralado, viajará
prop	proper name	América, Inglaterra, João, São Paulo
conj	conjunction	e, mas, mesmo, logo
det	determinant	a, os, um, umas
prep	preposition	com, de, sem, sobre
pro	pronoun	eu, estas, mim, isso
m	masculine	armário, ele, gato, touro
f	feminine	ela, gata, mesa, zebra
s	singular	barril, casa, este, flor
p	plural	barris, casas, estas, flores
1	first person	cantei, durmo, jogamos, veremos
2	second person	cantaste, dormes, jogais, vereis
3	third person	cantou, dorme, jogaram, verão
c1	1^st^ class conjugation	cantar, jogar, pescar, raspar
c2	2^nd^ class conjugation	comer, depor, por, viver
c3	3^rd^ class conjugation	dormir, sorrir, vestir, zumbir
ind	indicative	como, diria, deste, viajará
sub	subjunctive	coma, diga, desse, viajarmos
imp	imperative	coma, diga, demos, viajemos
pre	present	pego, tocas, olham, sabem
perf	preterit perfect	peguei, tocaste, olharam, souberam
imp	preterit imperfect	pegava, tocavas, olhavam, sabiam
fut	future	pegarei, tocarás, olharão, saberia
inf	infinitive	amar, beber, dormir, compor
ger	gerundive	amando, bebendo, dormindo, compondo
pp	past participle	amado, bebido, dormido, composto

Orthographic frequency column presents the raw frequency of the word form in the NILC, i.e., how many times this word appears in the entire NILC (e.g., *cachorro* = 397). Orthographic frequency per million shows the frequency of the word per one million words, which is the traditional frequency measure used in word-based corpora [2,12] (e.g., *cachorro* = 12.6523). Common logarithm (base 10) of the orthographic frequency presents the common logarithm of the raw orthographic frequency, which is used to linearize the corpora’s frequency distribution [5,6]. Zipf scale is a standardized measure of frequency, with the same interpretation in different corpora, like a Likert scale from 1 to 7[12] (e.g., *cachorro* = 4.1116). Zipf’s rank-frequency distribution is the rank position of the word in the whole corpus based on its frequency distribution and the Zipf’s law, that is, the most frequent word has rank 1, the second most frequent has rank 2, and so on, where words with the same frequency also have the same rank [5,6,41]. Number of letters displays the number of letters in the orthographic form (e.g., *cachorro* = 8) [49]. Number of homographs shows the number of words that have the same orthographic form but that belong to other grammatical categories (e.g., *ativa* ‘active’ = 3); then, homographs presents the grammatical categories of the other homograph words (e.g., *ativa* = adj, nom, ver) [4].

Orthographic uniqueness point column displays the position of the letter within the word, from left-to-right, at which this word becomes unique in the corpus, that is, the point that identifies the word unambiguously [48] (e.g., *cachorro* = 8). Orthographic neighborhood column presents the number of orthographic neighborhoods determined by the number of existing words in the corpus that can be matched by substituting one letter in any position at one time, i.e., with the Hamming distance of 1, according to Coltheart’s N [32,45] (e.g., *cachorro* = 2). Orthographic Levenshtein Distance presents the average of the Levenshtein distance among the 20 closest words; this measure incorporates words of different lengths in a graded and flexible measure of similarity (e.g., *cachorro* = 1.75), where the Levenshtein distance is the difference between two words in terms of the minimum number of additions, deletions, and substitutions required to change one word into the other [46,47].

CVCV orthographic structure column presents the CVCV structure for each word (e.g., *cachorro* = CVCCVCCV) [19]. Bigrams provides all of the bigrams for each word separated by the underline symbol ‘_’ and bounded by the hash symbol ‘#’ (e.g., *cachorro* = #c_ca_ac_ch_ho_or_rr_ro_o#); and trigrams presents all of the trigrams of each word (e.g., *cachorro* = #ca_cac_ach_cho_hor_orr_rro_ro#). Bigram frequency and trigram frequency columns display respectively the sum of the bigrams and trigrams frequencies that compose the word [18,50]. CVCV structure, bigrams, and trigrams information are largely used in language modeling and machine learning [36,37,51], as also for generating linguistic statistics about the corpus [5,18,19,35,50,52].

The reverse orthography column presents the reverse orthography from the orthographic column (e.g., *cachorro* = orrohcac). Reverse CVCV orthographic structure gives the reverse CVCV orthographic structure from the CVCV orthographic column (e.g., *cachorro* = VCCVCCVC). Reverse bigrams shows the reverse bigrams from the bigrams column (e.g., *cachorro* = #o_or_rr_ro_oh_hc_ca_ac_c#), and reverse trigrams presents the reverse trigrams from the trigrams column (e.g., *cachorro* = #or_orr_rro_roh_ohc_hca_cac_ac#). The random column gives each word a different random number between 0 and 1 with eight digits of precision, being useful for stimulus list randomization (e.g., *cachorro* = 0.46164741). Finally, the identity column shows the identity number of each word determined by its position in the lexicon organized by descending orthographic frequency and by orthography (a-z and 0–9).

### Internet site

The Brazilian Portuguese Lexicon was conceived as an easy, fast, and accessible Brazilian Portuguese word-based corpus with psycholinguistic, metalinguistic, and statistical information for everyone. We successfully fulfilled three very important criteria in developing the Brazilian Portuguese Lexicon: 1) we created a large, reliable, and complete database with information about Brazilian Portuguese words and lexicon, 2) we developed an instinctive and friendly interface between the user and the corpus, and 3) we offered free internet access, downloading of the full database, and specific search exporting.

To create the internet site, we imported to a MySQL database the whole corpus from a.txt file in which each lexical entry was placed on a different row and each column had a lexical information. The final corpus with 25 columns of information about the words and 215,175 rows with different entries (a 49 MB file) was hosted on a free server that fulfilled our requirements (i.e., <http://www.biz.nf/> offers: 250 MB space, 5 GB data transfer, 100 MB MySQL database, PHP 4/5, MySQL 5, POP3/SMTP webmail, FTP access, free host and free domain). We acquired the specific domain <http://www.lexicodoportugues.com/>, at which the Brazilian Portuguese Lexicon corpus is accessible, and linked it to our server. Then, we developed the internet page using the algorithm provided in **S3 Text**.

For the search engines and the results interface, we established some conventions that should be used when searching and interpreting the results. **Table 3** presents the grammatical categories (first seven rows) and grammatical information conventions [4]. The different information are separated with commas (e.g., *falas* ‘you speak’, gram_cat = ver, gram_inf = ind, pre, 2, sg).

### Search engines

**Fig 2:**
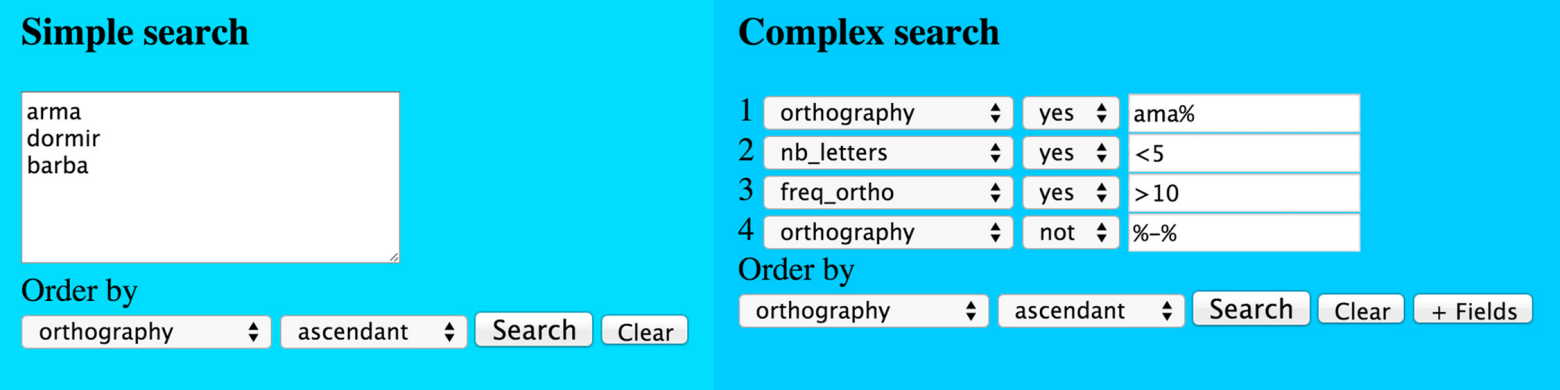
The simple and the complex input search engines of the Brazilian Portuguese Lexicon. Simple search allows a list of words as input and complex search allows specific criteria specification.

Inspired by the current psycholinguistic word-based corpora *Lexique* [4,48], CELEX [3], and ClearPOND [2], we constructed two types of search engines: 1) simple search and 2) complex search, as shown in **Fig 2**. The simple search engine allows the user to insert a list of words to search by directly typing in the text area or by copying and pasting data from other software; the words should be organized in different rows, separated by space or tabulation (Tab). The complex search allows the user to search words and/or groups of words by specifying and selecting different criteria. The first field specifies the column to be searched, the second field determines whether a criterion should be considered or not, and the third field is specified by the user. One special aspect of the search engines is that they allow the use of wildcards to search for partial chains of characters in the string fields and to search less than and greater than values in the numeric fields [4,48,53], according to the symbols listed in **Table 4**.

**Table 4:** Symbols used as wildcards in the search engines in the Brazilian Portuguese Lexicon.

Symbol	Function	Example	Result
_	substitute one or more character(s)	a_o_	amor, anos, aloe, após
%	substitute a chain of characters	am%	amor, ama, amei, amava
<	less than	nb_letters **<** 5	words with less than 5 letters
>	greater than	nb_homogr **>** 2	words with homographs greater than 2
< >	less than and greater than	freq_ortho **<** 10 **>** 6	words with orthographic frequency less than 10 and greater than 6

Both search engines include a space for selecting how the results should be displayed. The first field defines the column reference range, the second field specifies the direction (ascendant or descendant), the ‘Search’ button confirms the search, the ‘Clear’ button clears all fields, and the ‘+ Fields’ button makes available more fields for criterion insertion in the complex search [2,4]. Both search engines have an instinctive and friendly interface between the user and the corpus (see **Fig 2**), such that in the example the simple engine would search for the three words listed, and the complex engine would search for the orthography ‘ama%’ follow by an undefined chain of characters with less than five letters, frequency greater than 10, and with no hyphen in the orthography. Both engines would present the words in ascending order according to orthography.

### Search results

**Fig 3:**
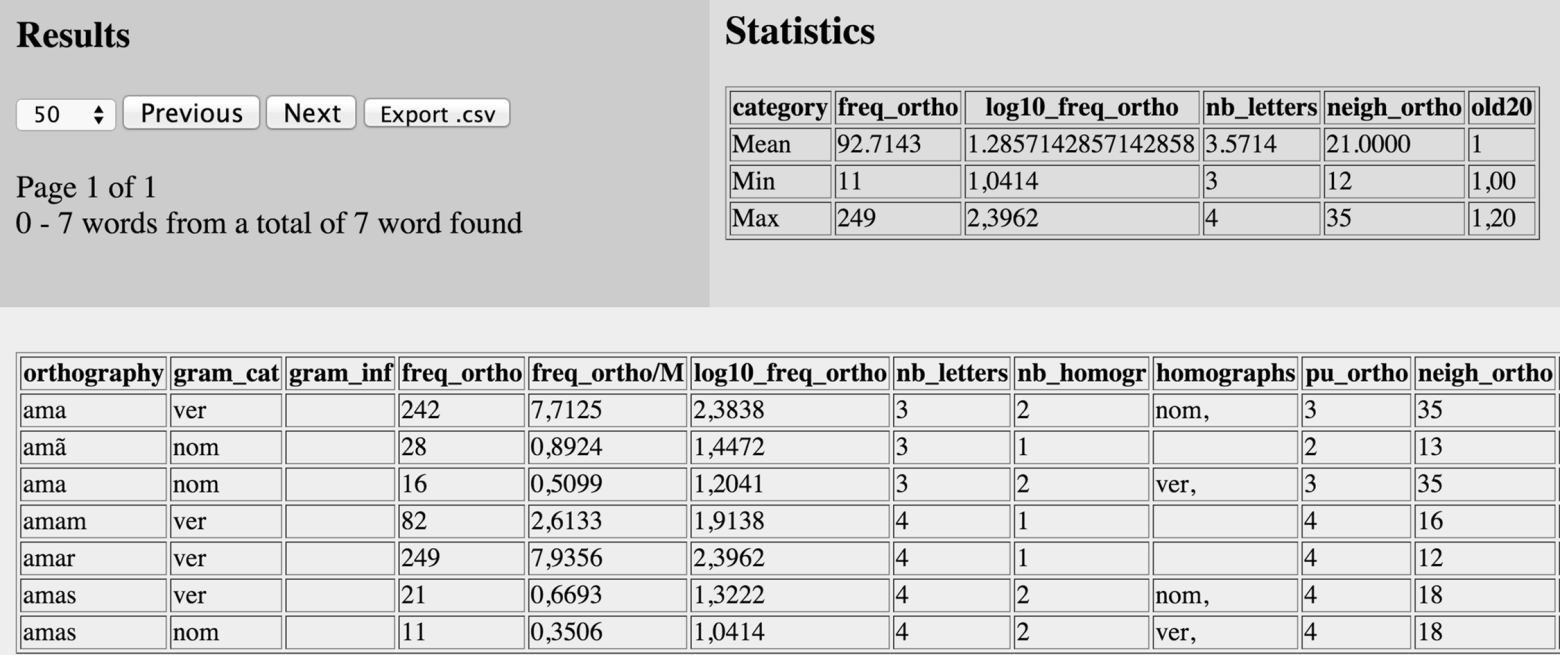
Results of the complex search made in the Fig 2 example. The top-left space presents the general search information, the top-right space provides basic statistics, and the down space displays the search results.

After a simple or complex search is completed by pushing the ‘Search’ button, the results are presented as shown in **Fig 3**. The top-left space presents a) the total number of pages, b) the page presented as the output, c) the range of entries presented on the page, and d) the total number of entries found. Furthermore, the user can select the number of entries to be displayed in the output, and there are two buttons for navigating between the result pages, i.e., the ‘Previous’ and ‘Next’ buttons. The user can also export the entire search as a downloadable.csv file by pushing the ‘Export.csv’ button [18,50,52].

The top-right space in **Fig 3** provides basic statistics calculated online from the current search. It presents the maximum, minimum, and mean values of the columns: a) ortho_freq, b) log10_ortho_freq, c) zipf_scale, d) bigram_freq, e) trigram_freq, f) nb_letters, g) ortho_neigh, and h) old20 [18,19]. The down space in **Fig 3** displays the results of the search. Each entry is organized on a different row, and the columns present the information about each entry. The results are arranged in ascending or descending order according to the column selected in the search engine; thus, users can easily control and manipulate the results to be presented and organized in line with their interests.

### Site resources: Downloads, Pseudowords, and Tools

We developed the rest of the internet site by inserting a header with the title of the corpus, a footer with the license and source information, and a sidebar with the links: a) Lexicon, b) Pseudowords, c) Downloads, d) Tools, e) Updates, f) Credits, and g) Statistical Linguistics. Lexicon sends the user to the main page; Pseudowords to the pseudowords generation engine; Downloads directs users to a page with downloadable files, such as the previously computed corpus, lists, tables, manual, and scripts; Tools provides information and links to related corpora, software, and literature; Updates presents the development history of the corpus; Credits describes the source of the original NILC, the developers of the Brazilian Portuguese Lexicon, and its license; and Statistical Linguistics provides a series of online tools for linguistic statistics. At this time, the Brazilian Portuguese Lexicon access is fully-available in the English and Portuguese languages, as also can be easily translated to other languages through the Google Translator plug-in in all pages.

During the development of the Brazilian Portuguese Lexicon, a great deal of information was generated and manipulated from the basic NILC source; many phenomena were noted, studied, and analyzed, including clitics, compound words, proper names, grammar, frequencies, letters, bigrams, trigrams, and neighborhoods. This information was carefully extracted, filtered, and organized from the full corpus to offer better comprehension and understanding of its general aspects [48], being made available as downloadable files on the Downloads page <http://www.lexicodoportugues.com/downloads_en.php/>.

Additionally, the Brazilian Portuguese Lexicon has a Brazilian Portuguese pseudoword generation engine <http://www.lexicodoportugues.com/pseudowords_en.php/> that creates pseudowords based on bigram and trigram structure and frequency as a function of grammatical categories [53]. Finally, the Brazilian Portuguese Lexicon provides a tool for statistical linguistics <http://www.lexicodoportugues.com/stat_ling_en.php/> that performs many statistical and metalinguistic functions [10,18,19,40,41,45,47,50,52].

### Creative Commons License

All of the materials and resources used to construct the Brazilian Portuguese Lexicon were free, with open access of the NILC from the *Linguateca* [17,38] and R software and packages [39–42]. After constructing the database and creating the internet page interface with the specific domain, we registered the Brazilian Portuguese Lexicon with a Creative Commons License <https://creativecommons.org/> of the Attribution-Non Commercial-ShareAlike 4.0 International type <http://creativecommons.org/licenses/by-nc-sa/4.0/>, which attributes to authors the appropriate credits, does not allow the use of the material for commercial purposes, and allows users to share, copy and redistribute the original material and to adapt, transform, and distribute the material under the same license as the original.

## Results

### The Brazilian Portuguese Lexicon

The final version of the Brazilian Portuguese Lexicon, which we called the Alpha version, is a word-based corpus with psycholinguistic and metalinguistic information. It was built from the POS-tagged NILC [17,38] and provides the most important information in the orthographic modality for stimuli selection, control and manipulation in psycholinguistic experiments [4,53,54], computational linguistic analysis [5,6], and language modeling [7,8,36,37,51]. Both search engines provide the essential characteristics for controlled search, such as copying and pasting, wildcard use, and range order control.

The user can easily navigate between the results, choosing the number of entries and the page to be displayed. Additionally, the entire results can be exported by using the ‘Export.csv’ button, or by selecting, copying (Ctrl+c), and pasting (Ctrl+v) the entries of interest into a different program [10,52]. Finally, the Brazilian Portuguese Lexicon provides basic statistics about the current search results [18,50].

### Lexicon distribution

**Fig 4:**
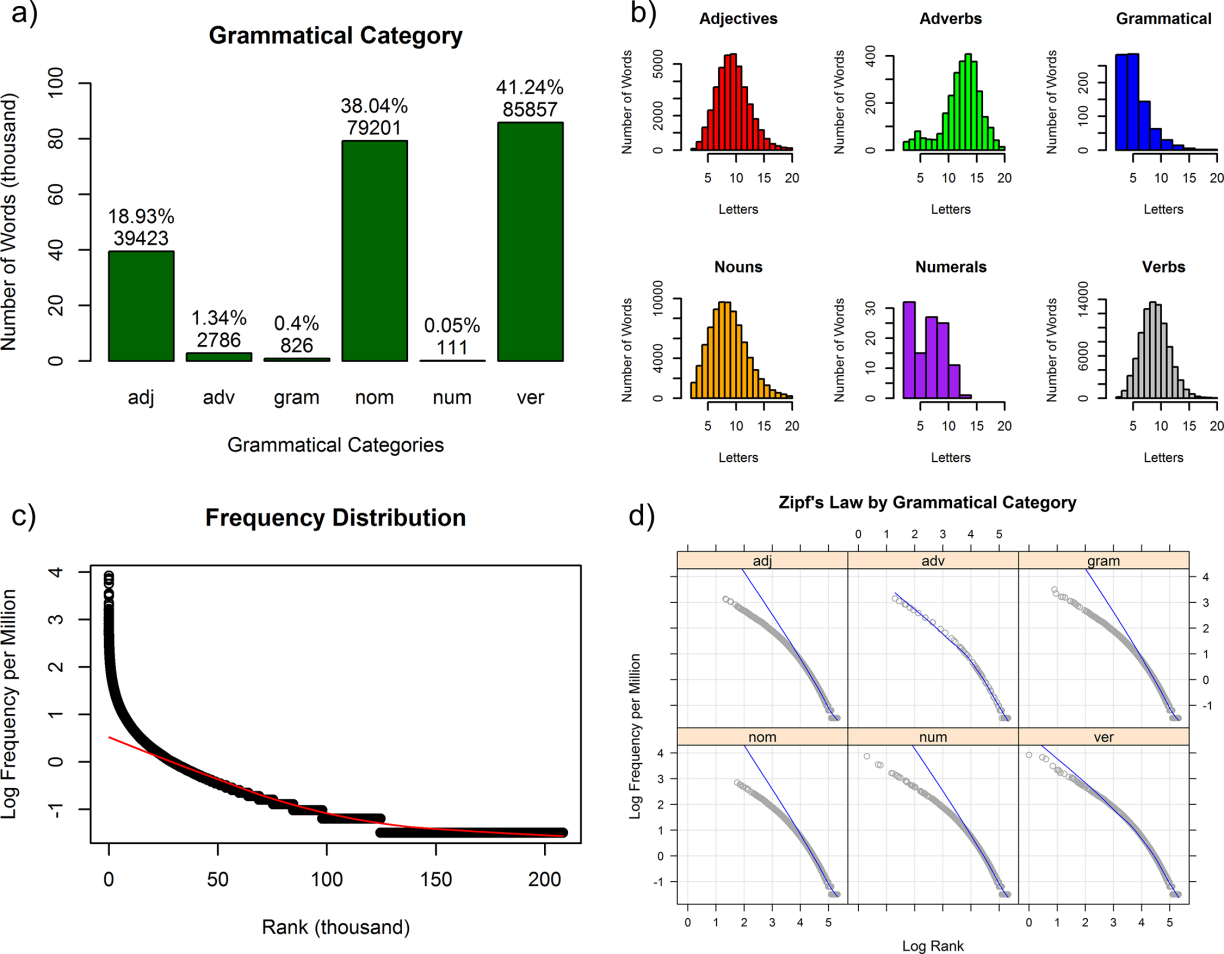
General distributions of the Brazilian Portuguese Lexicon corpus. a) number of words by grammatical category; b) number words according to the number of letters for each grammatical category; c) log10 frequency by word rank distribution; and d) Zipf’s law (i.e., log10 frequency by log10 rank) for each grammatical category.

**Fig 5:**
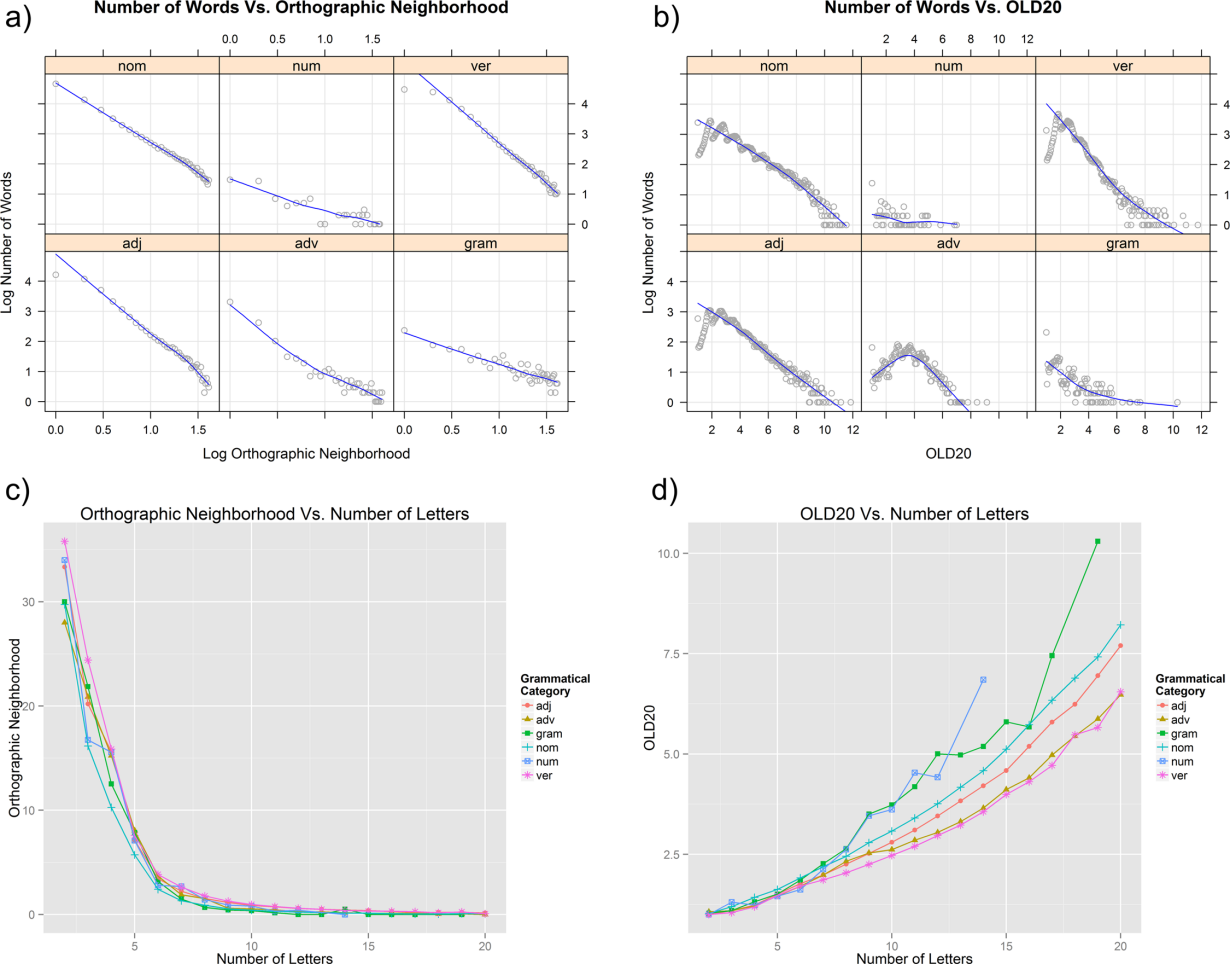
General interactions between variables in the Brazilian Portuguese Lexicon. a) log10 number of words by log10 orthographic neighborhood for each grammatical category; b) log10 number of words by OLD20 for each grammatical category; c) mean orthographic neighborhood by number of letters for each grammatical category; and d) mean OLD20 by the number of letters for each grammatical category.

With the Brazilian Portuguese Lexicon database and internet site ready for use, we analyzed its general distributions and the interactions between variables (i.e., informational columns) to provide an overall description and a global picture of the Brazilian Portuguese Lexicon. In **Fig 4**, we present the main general distributions; and in **Fig 5**, we present the variable interactions between grammatical categories, number of letters, orthographic neighborhood, OLD20, and number of words [2,53]. For a clear analysis, we applied basic filters to the full Brazilian Portuguese Lexicon data to eliminate extreme outliers and specific word information errors. We removed words with: a) more than 20 letters (.25%), b) more than 40 orthographic neighbors (.37%), c) more than 14 OLD20 (.01%), and d) more than 5 homographs (.03%), accounting for a total of .62% of the entire database. The general means and standard deviations according to grammatical category are shown in **Table 5**.

**Table 5:** General means and standard deviations between parentheses by grammatical category.

Gram. Cat.	Letters	Homographs	Ortho. PU	Ortho. N	OLD20
Adjectives	9.97(2.89)	1.24(0.47)	8.15(3.08)	1.57(3.19)	2.89(1.18)
Adverbs	12.82(3.39)	1.09(0.42)	7.22(2.88)	1.07(3.93)	3.47(1.12)
Grammatical	5.83(2.59)	1.55(0.86)	4.36(2.03)	8.37(10.47)	1.98(1.17)
Nouns	9.17(3.29)	1.12(0.35)	6.75(2.91)	1.81(4.44)	2.94(1.38)
Numerals	6.98(2.87)	1.61(0.89)	5.36(2.36)	6.43(9.59)	2.44(1.37)
Verbs	9.41(2.49)	1.08(0.29)	7.94(2.41)	1.86(3.29)	2.39(0.77)
**Total**	**9.45(2.96)**	**1.13(0.36)**	**7.51(2.82)**	**1.81(3.85)**	**2.71(1.16)**

Moving to the general distributions, **Fig 4A** shows that as expected, nouns and verbs are clearly the main categories, accounting for nearly 80% of the corpus, followed by adjectives, which account for less than half the number of nouns and verbs. Adverbs, grammatical and numerals represent only 1.79% of the corpus. Obviously, this asymmetric distribution is determined by the former’s open and latter’s closed grammatical categories and by the derivational and inflectional productivity within these categories [21,43,44]. Because verbs have a large inflectional paradigm in Portuguese, they account for the largest number of word types, whereas nouns are the main lexical category, comprising the highest number of lemmas. Adjectives, which are generally derived from nouns, are highly productive, whereas adverbs have low productivity, being derived from adjectives and/or nouns [22,44].

In **Fig 4B**, the ‘y’ axes in the different graphics represent the number of word limits in each grammatical category for better visualization of the specific distributions. Adjectives, nouns and verbs have more normal-like distributions as a function of the number of letters; these categories include the highest number of words between 7 and 8 letters. Adverbs have an almost normal-like distribution but with more words between 12 and 14 letters and with a left skewed tail of words with fewer than 8 letters. In contrast, grammatical words have a logarithmic-like distribution, with most words between 1 and 5 letters, whereas numerals show an irregular distribution.

**Fig 4C** presents the general word frequency distribution. Only a few words account for extremely high frequencies, and more than half of the corpus accounts for low frequencies [12]; the red line highlights the main smooth trend [6]. This logarithmic-like distribution matches perfectly with the corpora frequency distributions in other languages, such as Dutch, English, French, German and Spanish [2]. Next, in **Fig 4D**, the frequency distribution is plotted with both axes logarithmized according to grammatical category, and the blue lines highlight the main smooth trend, which allows for better linear visualization of Zipf’s law [5,6]. We observed that the adverbs and grammatical words have a large, extended distribution in the full corpus and are the most frequently occurring words, given that they appear in the first ranks. While closed categories can be easily fitted by a smooth line because of their low numbers of words occurring at low frequencies, the open categories include many words that accumulate at low frequencies, which explains their good fits at the low frequencies and, consequently, at the high ranks.

**Fig 5A and 5B** show the distributions of orthographic neighborhood and OLD20 densities, respectively, in the different grammatical categories. We noted that both measures of orthographic neighborhood show similar behaviors but that the traditional Coltheart’s N [32] must be plotted with a logarithmic ‘x’ axis for better linear visualization. Whereas high values in both measures have low numbers of words, low values for both measures have different behaviors, only the OLD20 shows a typical course in low OLD20, indicating that few words have no similar words. Coltheart’s N shows a general linear decrease, while OLD20 has a more complex spreading distribution [46]. Regarding the closed grammatical categories, we noted that orthographic neighborhood and OLD20 have lower densities and a more spreading distribution than open grammatical categories, which have a similar extended range in the corpus [5].

**Fig 5C and 5D** show the means of orthographic neighborhood and OLD20, respectively, for each grammatical category plotted as a function of the number of letters. We can easily observe the more logarithm-like distribution of the orthographic neighborhood Coltheart’s N [2,32] compared with the more graded linear-like distribution of the OLD20, as also the contrary nature of each measure [7,46]. We note that although both measures are functionally similar for short words (up to 5 letters), Coltheart’s N has limited utility, with practically no variation in longer words, as observed in the right tail in **Fig 5C**; in contrast, OLD20 measurement continues to be productive, even for long words, according to **Fig 5D**. More interestingly, Coltheart’s N presents small differences between the grammatical categories in short words and no differences in long words, but OLD20 presents grammatical category differences especially in long words, with a) closed categories presenting higher OLD20 for long words, b) adjectives and nouns presenting a similar behavior, with a higher OLD 20 for nouns, and c) adverbs and verbs presenting almost the same behavior. This analyses shows that the OLD20 is more sensitive to word length and has a larger range of coefficient distribution, better accounting for the word neighborhood in a guardedly flexible way; Coltheart’s N, meanwhile, is a more restricted and conservative measure of orthographic similarity [25,26,46,55].

### Comparisons to other corpora

**Fig 6:**
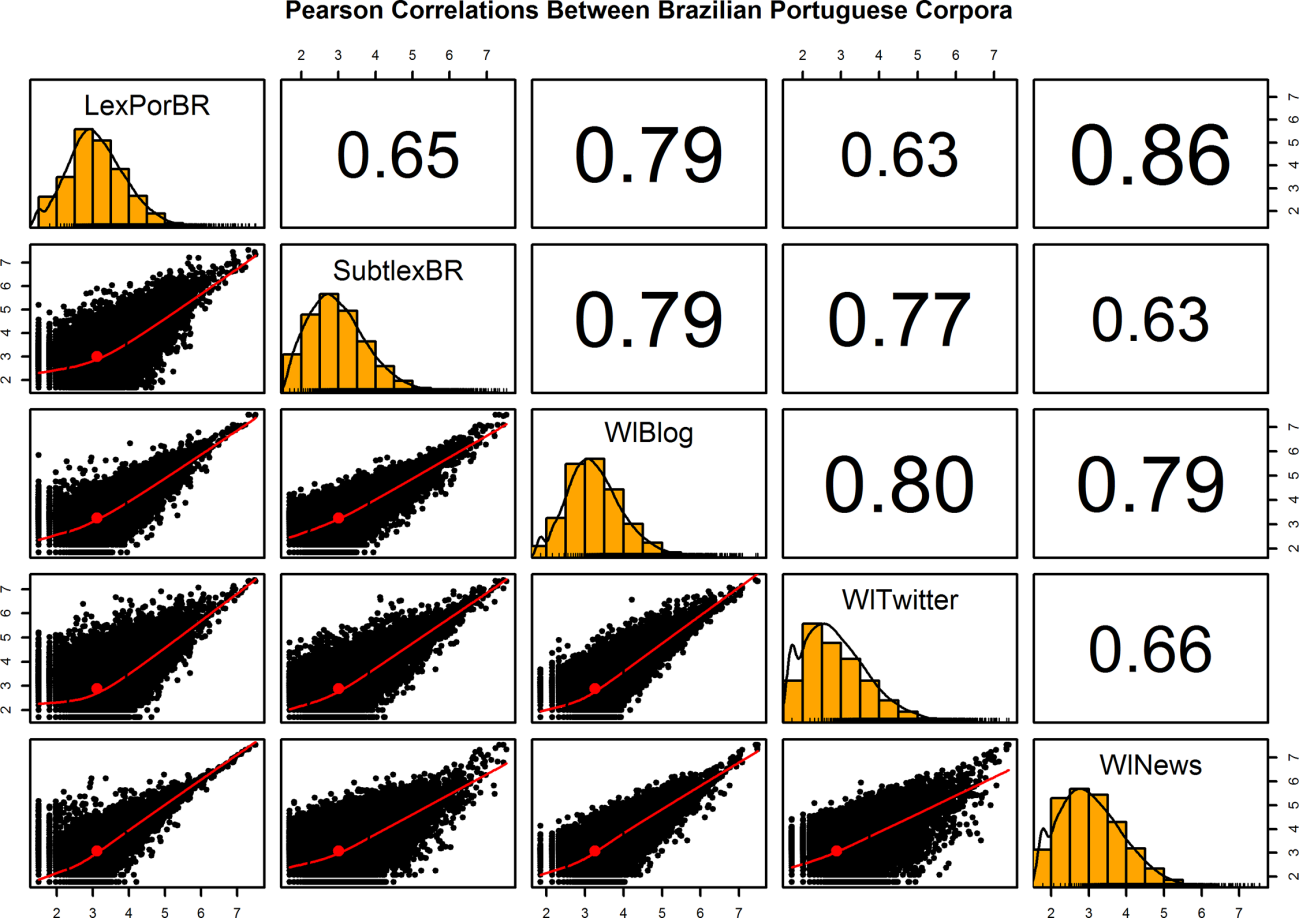
Correlations between the different current Brazilian Portuguese corpora. LexPorBR: Brazilian Portuguese Lexicon; SubtlexBR: SUBTLEX-PT-BR [15]; WlBlog, WlTwitter, and WlNews for the three Worldlex (Portuguese Brazil) corpora [16]. Correlations were calculated using the Zipf scale frequency [12]. Pearson correlation above the diagonal, histograms with corpora distribution on the diagonal, and bivariate scatter plots with loess smooth fits and ellipses below the diagonal [42].

In order to validate the Brazilian Portuguese Lexicon frequencies, we performed comparisons to the other Brazilian Portuguese corpora: a) SUBTLEX-PT-BR (136,147 word types) [15] and b) Worldlex (Portuguese Brazil) (191,795 total word types), which presents three corpora: blog (141,375 word types), Twitter (99,723 word types), and news (105,019 word types) [16]. Since these corpora do not present any POS-tagged information about grammatical categories, we summed the frequencies of the homographs in the Brazilian Portuguese Lexicon to account for orthographic form total frequencies, resulting in a total of 202,167 word types. Therefore, we analyzed the corpora through Pearson correlations using the Zipf scale [12] of the 45,968 word types that were common to the different corpora and that have frequency above 0 [13,14], as shown in **Fig 6**. The high correlations between the Brazilian Portuguese Lexicon and the other corpora validate its frequency distribution which is comparable to the other corpora. As expected, the correlation differences are in function of the textual genres and language modality present in each corpus, while the highest correlations were between the Brazilian Portuguese Lexicon, WlBlog, and WlNews, the lowest correlation was between the Brazilian Portuguese Lexicon and the WlTwitter.

Thus, we tested the significance of the difference between the correlations of the Brazilian Portuguese Lexicon and other corpora using Fisher r-to-z transformations [12,15,42]: LexPorBR/SubtlexBR vs. LexPorBR/WlBlog z = 45.89, LexPorBR/SubtlexBR vs. LexPorBR/WlTwitter z = 5.76, LexPorBR/SubtlexBR vs. LexPorBR/WlNews z = 79.69, LexPorBR/WlBlog vs. LexPorBR/WlTwitter z = 51.65, LexPorBR/WlBlog vs. LexPorBR/WlNews z = 33.81, LexPorBR/WlTwitter vs. LexPorBR/WlNews z = 85.45. All correlations and difference between correlations were highly significant (p < .0001). While the Brazilian Portuguese Lexicon significantly correlated with all other corpora, the correlations were also significantly different between them, putting in evidence the differences between the WlTwitter and WlNews, and the SubtlexBR and WlNews, with the highest z-values; as also the similarities between the SubtlexBR and WlTwitter, with the lowest z-values. These results underlines the informal and conversational aspects of the SubtlexBR and WlTwitter, and the formal and written aspects of the Brazilian Portuguese Lexicon, WlBlog, and WlNews [16].

Afterwards, we investigated the relative percentage of word types missing in each two corpora [14]. For this aim, we divided the number of common word types in each two corpora by the number of word types from the corpora of interest, according to **Table 6**. This table should be read as the top-head corpus contains the percentage of the left column corpus, and the left column corpus is contained by the top-head corpus. The Brazilian Portuguese Lexicon systematically yields higher values of word types contained than other corpora, presenting evidence as being the most complete corpus, with larger and more distributed word types.

**Table 6:** Relative percentage (%) of word types contained in the LexPorBR, SubtlexBR [15], and Worldlex (Portuguese Brazil) [16] corpora. The head corpus contains the percentage of word types of the left corpus and the left corpus is contained by the head corpus.

	LexPorBr	SublexBR	WlBlog	WlTwitter	WlNews
**LexPorBR**	100	46.39	34.14	32.13	23.17
**SubtlexBR**	63.89	100	52.26	42.57	23.13
**WlBlog**	53.94	50.43	100	26.93	26.04
**WlTwitter**	66.52	57.93	48.46	100	40.57
**WlNews**	60.09	60.09	45.06	37.41	100

Finally, we were interested to know which most frequent words were overestimated and underestimated [11] by the Brazilian Portuguese Lexicon in relation to the other corpora. Overestimated words are those that are present in the Brazilian Portuguese Lexicon, but are not present in the SUBTLEX-PT-BR or Worldlex (Portuguese Brazil); underestimated words are those that are not present in the Brazilian Portuguese Lexicon, but are present in the SUBTLEX-PT-BR or Worldlex (Portuguese Brazil), as shown in **Table 7**. We avoided proper names, and hyphenated composed words and verbs with clitics. These words were manually and carefully verified by two Brazilian Portuguese native-speakers. We note that the Brazilian Portuguese Lexicon presents common words as overestimated ones and specific words as underestimated ones; most part of the underestimated words are diminutives in the Worldlex (Portuguese Brazil) and inflected verbs in the SUBTLEX-PT-BR, differently, the overestimated ones are common words, as the object pronouns *te* ‘you’ and *se* ‘him/her/it’, the adverb *tão* ‘so’, the indefinite pronoun *tudo* ‘all’, and the inflected auxiliary verb *teve* ‘he/she/it had’ [43,44]. We remark that it was necessary to verify a large number of words in the SUBLETX-PT-BR and Worldlex (Portuguese Brazil) to complete the lists because these corpora present a lot of proper names, foreign language words, and words with accentuation and orthographic errors; also, these words are in lower Zipf scale ranges.

**Table 7:** Overestimated and underestimated words by the Brazilian Portuguese Lexicon compared to the SUBTLEX-PT-BR [15] and Worldlex (Portuguese Brazil) [16]. Between parentheses is the number of the most frequent words verified to list the 10 words presented in each list; Zipf scale range interval of the words found is indicated under heads.

Overestimated SubtlexBR (116) 5.77–4.59	Underestimated SubtlexBR (1343) 4.16–3.62	Overestimated Worldlex (125) 5.77–4.54	Underestimated Worldlex (264) 4.44–3.82
tão	matarei	tão	esmaltes
te	meritíssimo	te	presencial
se	danar	se	medite
cola	consegues	cola	disponibilizados
tudo	estrague	tudo	empreendendorismo
teve	abaixem	teve	tadinho
cambial	larguem	porte	solzinho
verdadeiro	percebes	forço	viciei
porte	esperaremos	vôo	lindinho
petista	odeie	colher	quitosana

## Discussion

As is the case with many psycholinguistic word-based corpora in different languages, e.g., English, MRC [9]; French, *Lexique* [4]; Spanish, *BuscaPalabras* [10]; Dutch, English, and German, CELEX [3]; and Dutch, English, French, German, and Spanish, ClearPOND [2]; the Brazilian Portuguese Lexicon aims to fill a gap in the descriptive lexical information about Brazilian Portuguese. Word-based corpora are extremely useful for selecting experimental stimuli in psycholinguistic experiments [24,27,28,30,34,54], computational linguistics [5,6], lexicon projects that use large number of stimuli, such as American English [1], British English [55], French [25], and Dutch [26], and in language modeling and simulation [7,8,35–37,51].

In this first Alpha version of the Brazilian Portuguese Lexicon, all of the orthographic information is provided, greatly facilitating the selection, manipulation, control, and analysis of the words as stimuli for psycholinguistic research. We perceived that the lack of this type of description and lexical instrument in Brazilian Portuguese critically complicated the selection of stimuli in psycholinguistic experiments. In this sense, the Brazilian Portuguese Lexicon will strongly contribute to psycholinguistic research in Brazilian Portuguese, such as, for example, that of [31], who researched the orthographic neighborhood effects, and that of [33], who investigated the representation of gender-inflected words using small, non-standard, and non-controlled corpora for their stimuli selection. The Brazilian Portuguese Lexicon provides the orthographic neighborhood Coltheart’s N [32], the Orthographic Levenshtein Distance among the 20 closest words [46], the Zipf scale [12], the grammatical category, the number of homographs, and the bigram and trigram frequencies, along with the most important lexical information described above [3,6,19,54].

Furthermore, the absence of a robust, referential, reliable, and friendly instrument like the Brazilian Portuguese Lexicon could likely impede, restrain, and demotivate psycholinguistic, computational linguistic, and language modeling research in Brazilian Portuguese because these fields require stimuli with standard controls, information, and specific characteristics. For example, orthographic recognition has been studied from different perspectives, resulting in the development of different visual recognition models [7,54]. The Brazilian Portuguese Lexicon might also contribute to language modeling, learning, and simulation in Brazilian Portuguese [7,8,37], such as visual word recognition [51], word length [49], orthographic neighborhood [32], word frequency [24], agrammatism [29], and multilingualism [28].

One important aspect of the Brazilian Portuguese Lexicon is that it can be accessed at no charge on the internet, which means that anyone in any place with internet has free access to the complete corpus and all of the resources available [2]. Additionally, the complete corpus can be downloaded and saved at the Downloads page to be used offline in a spreadsheet or text software [48]. The Brazilian Portuguese Lexicon differs from the majority of other corpora by delivering a module in the results that provides general statistics about the search [19], with the maximum, minimum, and mean values of a) ortho_freq, b) log10_ortho_freq, c) zipf_scale, d) bigram_freq, e) trigram_freq, f) nb_letters, g) ortho_neigh, and h) old20 [10,18,50,52,53].

Regarding the Brazilian Portuguese Lexicon distributions, the general frequency distribution is perfectly compatible and comparable with word-based corpora from other languages [2]. The main grammatical categories (i.e., adjectives, nouns, and verbs) comprise more than 98% of the corpus and presents normal-like distributions as a function of number of letters; differently, adverbs, grammatical, and numerals represent less than 2% of the corpus, presenting idiosyncratic distributions. Furthermore, open grammatical categories present interesting differences when compared to closed ones regarding the orthographic neighborhood and OLD20. Also, the orthographic neighborhood and OLD20 present particular behaviors as a function of the number of letters [2], with the OLD20 being a more graded measure than Coltheart’s N for word similarity [46,47].

Moving to the comparisons of the Brazilian Portuguese Lexicon to the SUBTLEX-PT-BR [15] and Worldlex (Portuguese Brazil) [16], our analysis were based on the Zipf scale [12], which is a standardized frequency measure. This scale provides a clear intuition about the range of frequencies in different corpora; while Zipf scale values below 3 are low-frequency words, values above 4 are high-frequency words, values above 6 are very high-frequency words, and only few grammatical words have values above 7.

It was a surprise to found that only 45,968 word types overlap between the different corpora (LexPorBR, SubtlexBR, WlBlog, WlTwitter, and WlNews), but later analysis clearly revealed that this result was provoked by a large number of words from foreign languages, proper names, and words with accentuation and orthographic errors in the SUBTLEX-PT-BR and Worldlex (Portuguese Brazil). Clearly, the WlTwitter presents the most informal language, with many errors of orthography and accentuation; as also the most restricted lexicon, as evidenced by the largest word tokens (19.5 million) and lowest word types (99,723) relation in the Worldlex (Portuguese Brazil) [16].

The high and significantly Pearson correlations with the SUBTLEX-PT-BR and Worldlex (Portuguese Brazil) validated the Brazilian Portuguese Lexicon frequency distributions as a large, diversified, stable, and reliable corpus [15,16]. As expected, in function of the textual genres and language modality present in each corpus, the Brazilian Portuguese Lexicon presented the highest correlations with the WlBlog and WlNews, as well as the lowest correlation with the WlTwitter. The correlation comparisons were still significantly different and put in evidence the differences between the corpora; while the SUBTLEX-PT-BR and WlTwitter provide more informal, conversational, and phonological frequencies, the Brazilian Portuguese Lexicon, WlBlog, and WlNews provide more formal, narrative, and orthographic frequencies [16].

Our analyses on overestimated and underestimated words showed clearly that the Brazilian Portuguese Lexicon is the most complete corpus with a large, variated, and distributed range of word type when compared to the other corpora. Most important, while most part of the Brazilian Portuguese Lexicon underestimated words are diminutive words (all words in Portuguese have a potential diminutive) and inflected verbs (Portuguese has a large verbal inflectional paradigm), the overestimated words are very common words, as pronouns, adverbs, and auxiliary verbs [43,44].

In a striking way, the manual verification performed by the Brazilian Portuguese native-speakers and language manuals [21,43,44] showed that the most underestimated words from the SUBTLEX-PT-BR are English (discussed in [15]) and Spanish words (i.e., Spanish composed words and verbs with clitics are directly concatenated, while Portuguese uses hyphen [21,22], e.g., Portuguese: *sentar-se*, Spanish: *sentarse* ‘to sit’). Further scrutiny in this corpus showed that there is no hyphenated word listed, but the composed words and verbal clitics are directly concatenated, which makes all these forms invalid forms of the Portuguese language [21,22,43,44]. This finding put serious limitations on the usability of the SUBTLEX-PT-BR [15]. The same kind of artifact was found in the Worldlex (Portuguese Brazil) [16], however, it might be that internet users sometimes do not employ hyphen in blog and Twitter fast typing. It highlights the requirement and importance of native-speaker verification and POS-tag analysis [12–14] in the computation of subtitle and internet corpora.

One can also notice that SUBTLEX-PT-BR and Worldlex (Portuguese Brazil) include a large number of proper names (which could be considered noise in the corpus, since psycholinguistic research is not generally interested in proper names), as evidenced by the large number of words verified to achieve the 10 most underestimated words in each corpus; the filtering of these proper names would probably reduce significantly the number of word types in both corpora. As expected, the 10 underestimated words by the Brazilian Portuguese Lexicon were found in a Zipf scale range lower than the overestimated words [12]. Differently, the Brazilian Portuguese Lexicon offers a file containing 293,198 entries in the Downloads page with the definition ‘proper name’ in the grammatical category; indeed, these proper names are much more Brazilian Portuguese related than subtitles translation and internet proper names.

It is interesting to note that corpora based on subtitles have provided amazing results as frequency predictors on lexical processing, especially in lexical decision and word naming tasks [1,11–14,25,26,55]. Also, subtitle and internet corpora are extremely interesting for language evolution, neologisms, textual genres, typing and spelling errors, and informal language analysis. Nevertheless, according to Cai and Brysbaert (2010, p.4) [14] “criticisms can be raised against films as a representative source of language (they often depict American situations, are biased towards certain topics such as police investigations, do not include everything that is said, the language is not completely spontaneous, etc.)”. Indeed, subtitle corpora presents mostly dialogue genre, oral modality words, and are adapted in translated films, which could be considered a phonological database, while written corpora could be considered an orthographic database, that is, the words used in written modality, as differentiated in the *Lexique* [4]. Otherwise, when looking for and selecting stimuli for psycholinguistics experiments, researches are often not interested in abbreviations, orthographic and accentuation errors, slangs, foreign language words, bad words, and words that are not used in the written modality, but they are looking for established, correct spelling, and specific target words according to their conditions and hypothesis (i.e., masculine/feminine, singular/plural, present/past, regular/irregular, rhyming, prosody, semantic field, etc.) [34,53]. Moreover known words do not always appear in corpora made from speech. For example, if somebody is researching the oral obsoleted French tenses *passé simple* or *imperfait subjonctif*, or more formal and specific words, the subtitle or internet corpora will be very limited, and words must be selected from orthographic corpora being determined by large and varied written literature and news. Even more, one of the overestimated words by the Brazilian Portuguese Lexicon in both other corpora was the established word *petista* ‘person from the PT political party’, which would be never found in subtitle films, but probably researches have a lot of interest in study this word as a marker of morphological productivity [21].

In this sense, researches sometimes want to select words that are not in subtitle and internet corpora, and do not want to select many words that are in these corpora. Therefore, it seems that phonological and orthographic corpora are complementary; in the way that different modalities can help and explain each other based on language frequency phenomena [14]. Also, researches are interested in having access to as many metalinguistic and psycholinguistic information as possible instantaneously for stimuli and condition matching for objective purposes on their hypothesis [6,34].

Therefore, the Brazilian Portuguese Lexicon corpus presents useful characteristics in terms of its size, accessibility, speed of use, and information about the Brazilian Portuguese lexicon. The sections above described the general features of the Brazilian Portuguese Lexicon, analyzed the general distributions and interactions of different variables, and compared to the other current Brazilian Portuguese databases SUBTLEX-PT-BR [15] and Worldlex (Portuguese Brazil) [16], confirming that most characteristics of the Brazilian Portuguese Lexicon are in perfect harmony with the other useful psycholinguistic word-based corpora [2–4], and is superior than the current Brazilian Portuguese databases SUBTLEX-PT-BR [15] and Worldlex (Portuguese Brazil) [16] when regarding a) number of word types, b) number of metalinguistic and psycholinguistic information provided, c) Pearson correlation to other corpora, d) POS-tagged information, e) control of foreign language words, f) control of proper names, g) internet site and interface facilities, h) downloads available, i) corpus origin, j) pseudoword generation engine, k) statistical linguistic tools, and l) free and open-code access.

Finally, we believe that we have developed synthetic, objective, and logical algorithms for constructing and developing the Brazilian Portuguese Lexicon in R software [39] and for the HTML, PHP, and MySQL interface of the main internet page. These scripts are available in **S2 Text** and **S3 Text**, respectively. They can be easy implemented, adapted, and modified for different corpora, uses, and purposes.

## Final Remarks and Developments

In this article, we presented the psycholinguistic word-based corpus: Brazilian Portuguese Lexicon, *Léxico do Português Brasileiro (LexPorBR)*. It is a stable, reliable, and complete database that we strongly believe will have long-term utility, meeting all needs for psycholinguistic, computational linguistic, and language modeling research in Brazilian Portuguese, as also multilingualism and cross-linguistic research [2,28]. The Brazilian Portuguese Lexicon was constructed from the NILC [38]; it contains more than 30 million word tokens, 215 thousand word types, 160 thousand lemmas, 290 thousand proper names, and 25 columns of information; it is accessible free of charge on the internet; and it provides a basic pseudoword generation engine [53], as also a statistical module in the results [18]. The Brazilian Portuguese Lexicon is also available for download in the **S1 File**. We called this first version of the Brazilian Portuguese Lexicon the Alpha version; the Beta version will expand on the available information and will contain phonological and syllabic forms and, consequently, all of the derived phonological and syllabic categories [2,4,19,56,57], providing more information and possibilities in the corpus searches. Indeed, the Brazilian Portuguese Lexicon words will be compared with Brazilian Portuguese dictionaries to develop more selective and restrictive criteria about existing words, grammatical categories, grammatical and semantic information.

Finally, the Brazilian Portuguese Lexicon has a basic Brazilian Portuguese pseudoword generation engine that creates pseudowords based on bigram and trigram structure and frequency as a function of the grammatical categories, and we are working with different types of pseudoword engines based on our corpus data [53,58,59]. In addition, we made available an online linguistic tool that performs many statistical and metalinguistic functions and that will be further developed.

## Supporting Information

S1 Text Orthographic conventions. Relation between orthography and conventions: ‘V’ for vowel, ‘C’ for consonant, ‘P’ for punctuation, ‘A’ for accent, ‘S’ for symbol, and ‘N’ for number.
(DOCX)

S2 Text R algorithm. Algorithm in R software for the Brazilian Portuguese Lexicon development and construction.
(DOCX)

S3 Text Brazilian Portuguese Lexicon main page algorithm. Algorithm in HTML, CSS, Java, MySQL, and PHP programming languages of the Brazilian Portuguese Lexicon main page (index_en.php).
(DOCX)

S1 File Brazilian Portuguese Lexicon ZIP file. ZIP file containing the complete Brazilian Portuguese Lexicon in.txt file with the columns separated by tabulation (Tab).
(ZIP)
