# Supplementary material for: The Brazilian Portuguese Lexicon: An Instrument for Psycholinguistic Research
Source: PLoS One. 2015 Dec 2;10(12):e0144016. doi: 10.1371/journal.pone.0144016 (PMC4668042; doi:10.1371/journal.pone.0144016)
Supplement: S1 Text — Relation between orthography and conventions: ‘V’ for vowel, ‘C’ for consonant, ‘P’ for punctuation, ‘A’ for accent, ‘S’ for symbol, and ‘N’ for number. (DOCX) [file pone.0144016.s001.docx]

The Brazilian Portuguese Lexicon: An Instrument for Psycholinguistic Research

Gustavo L. Estivalet^1,2*^ and Fanny Meunier^1,2^

^1^CNRS UMR5304, Laboratoire sur le Langage, le Cerveau et la Cognition, Institut de Sciences Cognitives, Bron, France

^2^Université Claude Bernard Lyon 1, Université de Lyon, Lyon, France

***Corresponding author:** Gustavo Lopez Estivalet, Laboratoire sur le Langage, le Cerveau et la Cognition, Institut de Sciences Cognitives, Bron, France. Phone: +33 651 231 584. E-mail: [gustavo.estivalet@isc.cnrs.fr](mailto:gustavo.estivalet@isc.cnrs.fr) (GLE).

# Supporting Information

**S1 Text. Orthographic conventions.** Relation between orthography and conventions: ‘V’ for vowel, ‘C’ for consonant, ‘P’ for punctuation, ‘A’ for accent, ‘S’ for symbol, and ‘N’ for number.

| **Orthography** | **Convention** |
| --- | --- |
| a | V |
| e | V |
| i | V |
| o | V |
| u | V |
| á | V |
| à | V |
| â | V |
| ã | V |
| ä | V |
| é | V |
| è | V |
| ê | V |
| ë | V |
| í | V |
| ì | V |
| î | V |
| ï | V |
| ó | V |
| ò | V |
| ô | V |
| õ | V |
| ö | V |
| ú | V |
| ù | V |
| û | V |
| ü | V |
| y | V |
| b | C |
| c | C |
| ç | C |
| d | C |
| f | C |
| g | C |
| h | C |
| j | C |
| k | C |
| l | C |
| m | C |
| n | C |
| p | C |
| q | C |
| r | C |
| s | C |
| t | C |
| v | C |
| w | C |
| x | C |
| z | C |

| **Orthography** | **Convention** |
| --- | --- |
| 0 | N |
| 1 | N |
| 2 | N |
| 3 | N |
| 4 | N |
| 5 | N |
| 6 | N |
| 7 | N |
| 8 | N |
| 9 | N |
| ´ | A |
| ` | A |
| ^ | A |
| ~ | A |
| ¨ | A |
| , | P |
| ; | P |
| . | P |
| : | P |
| ? | P |
| ! | P |
| - | P |
| & | S |
| @ | S |
| § | S |
| # | S |
| % | S |
| $ | S |
| £ | S |
| ¤ | S |
| * | S |
| + | S |
| = | S |
|  | S |
| < | S |
| > | S |
| { | S |
| } | S |
| [ | S |
| ] | S |
| ( | S |
| ) | S |
| / | S |
| \ | S |
| " | S |
| \| | S |
| _ | S |
| ° | S |
