# Supplementary material for: The Brazilian Portuguese Lexicon: An Instrument for Psycholinguistic Research
Source: PLoS One. 2015 Dec 2;10(12):e0144016. doi: 10.1371/journal.pone.0144016 (PMC4668042; doi:10.1371/journal.pone.0144016)
Supplement: S2 Text — Algorithm in R software for the Brazilian Portuguese Lexicon development and construction. (DOCX) [file pone.0144016.s002.docx]

The Brazilian Portuguese Lexicon: An Instrument for Psycholinguistic Research

Gustavo L. Estivalet^1,2*^ and Fanny Meunier^1,2^

^1^CNRS UMR5304, Laboratoire sur le Langage, le Cerveau et la Cognition, Institut de Sciences Cognitives, Bron, France

^2^Université Claude Bernard Lyon 1, Université de Lyon, Lyon, France

***Corresponding author:** Gustavo Lopez Estivalet, Laboratoire sur le Langage, le Cerveau et la Cognition, Institut de Sciences Cognitives, Bron, France. Phone: +33 651 231 584. E-mail: [gustavo.estivalet@isc.cnrs.fr](mailto:gustavo.estivalet@isc.cnrs.fr) (GLE).

# Supporting Information

**S2 Text. R algorithm.** Algorithm in R software for the Brazilian Portuguese Lexicon development and construction.

## Install packages

install.packages("languageR")

install.packages("vwr")

## Load packages

library(languageR)

library(vwr)

## Functions

## Reverse string function

strrev <- function(x) sapply(lapply(strsplit(x, NULL), rev), paste, collapse="")

## Read file (ortho and ortho_freq)

form.adj <- read.table(file = "C:/…/list.forms.ADJ.txt", sep = "\t", header = FALSE)

form.adv <- read.table(file = "C:/…/list.forms.ADV.txt", sep = "\t", header = FALSE)

form.gram <- read.table(file = "C:/…/list.forms.GRAM.txt", sep = "\t", header = FALSE)

form.nom <- read.table(file = "C:/…/list.forms.N.txt", sep = "\t", header = FALSE)

form.num <- read.table(file = "C:/…/list.forms.NUM.txt", sep = "\t", header = FALSE)

form.ver <- read.table(file = "C:/…/list.forms.V.txt", sep = "\t", header = FALSE)

## Grammatical category (gram_cat)

form.adj$gram_cat <- "adj"

form.adv$gram_cat <- "adv"

form.gram$gram_cat <- "gram"

form.nom$gram_cat <- "nom"

form.num$gram_cat <- "num"

form.ver$gram_cat <- "ver"

## Merge files

form1 <- merge(form.adj, form.adv, form.gram, form.nom, form.num, form.ver, all = TRUE)

head(form1)

## Define column names

colnames(form1) <- c("ortho_freq", "ortho", "gram_cat")

head(form1)

## As character

form1$ortho <- as.factor(form1$ortho)

form1$gram_cat <- as.factor(form1$gram_cat)

str(form1)

## Total rows 1

total.nrow1 <- nrow(form1)

total.nrow1

## Total frequency 1

total.freq1 <- sum(form1$ortho_freq)

total.freq1

## Grammatical category rows 1

cat.nrow1 <- xtabs(~gram_cat, data = form1)

cat.nrow1

## Grammatical category frequency 1

cat.freq1 <- xtabs(ortho_freq~gram_cat, data = form1)

cat.freq1

## Order orthography

form1 <- form1[order(form1$ortho),]

head(form1)

## Lower cases

form2$ortho <- tolower(form2$ortho)

head(form2)

## Aggregate uppercases and lowercases

form2 <- aggregate(form2$ortho_freq, list(form2$ortho, form2$gram_cat), sum)

colnames(form2) <- c("ortho", "gram_cat", "ortho_freq")

head(form2)

## Total rows 2

total.nrow2 <- nrow(form2)

total.nrow2

total.nrow2.lost <- 100 * (1 - total.nrow2/total.nrow1)

total.nrow2.lost

## Total frequency 2

total.freq2 <- sum(form2$ortho_freq)

total.freq2

total.freq2.lost <- 100 * (1 - total.freq2/total.freq1)

total.freq2.lost

## Grammatical category rows 2

cat.nrow2 <- xtabs(~gram_cat, data = form2)

cat.nrow2

cat.nrow2.lost <- 100 * (1 - cat.nrow2/cat.nrow1)

cat.nrow2.lost

## Grammatical category frequency 2

cat.freq2 <- xtabs(ortho_freq~gram_cat, data = form2)

cat.freq2

cat.freq2.lost <- 100 * (1 - cat.freq2/cat.freq1)

cat.freq2.lost

## Order frequency and orthography

form2 <- form2[order(form2$ortho_freq, form2$ortho, decreasing = TRUE),]

head(form2)

## Number letters (nb_letters)

form2$nb_letters <- nchar(form2$ortho)

head(form2)

## Remove number of letters > 30

form3 <- subset(form2, nb_letters < 31)

form3 <- droplevels(form3)

## Total rows 3

total.nrow3 <- nrow(form3)

total.nrow3

total.nrow3.lost <- 100 * (1 - total.nrow3/total.nrow2)

total.nrow3.lost

## Total frequency 3

total.freq3 <- sum(form3$ortho_freq)

total.freq3

total.freq3.lost <- 100 * (1 - total.freq3/total.freq2)

total.freq3.lost

## Grammatical category rows 3

cat.nrow3 <- xtabs(~gram_cat, data = form3)

cat.nrow3

cat.nrow3.lost <- 100 * (1 - cat.nrow3/cat.nrow2)

cat.nrow3.lost

## Grammatical category frequency 3

cat.freq3 <- xtabs(ortho_freq~gram_cat, data = form3)

cat.freq3

cat.freq3.lost <- 100 * (1 - cat.freq3/cat.freq2)

cat.freq3.lost

## Identification (id)

form3$id <- c(1:nrow(form3))

head(form3)

## Frequency per million (ortho_freq_M)

form3$ortho_freqM <- 1000000 * form3$ortho_freq/total.freq3

head(form3)

## Log10 orthographic frequency (log10_ortho_freq)

form3$log10_ortho_freq <- log10(form3$ortho_freq)

head(form3)

## Zipf scale (zipf_scale)

form3$zipf_scale <- log10(form3$freq_orthoM) + 3

head(form3)

## Zipf's rank (zipf_rank)

zr <- data.frame(xtabs(~ortho_freq, data = form3))

zr <- zr[nrow(zr):1,]

zr$zipf_rank <- cumsum(zr$Freq)

zr <- zr[,c(1,3)]

form3 <- merge(form3, zr)

## Aggregate homographs

form3$dummy <- 1

nb_homogr <- aggregate(form3$dummy, list(form3$ortho), sum)

colnames(nb_homogr) <- c("ortho", "nb_homogr")

form4 <- (merge(form3, nb_homogr, by = "ortho"))

head(form4)

#### Homographs ####

#### Letters ####

#### CVCV structure ####

#### Bigrams ####

#### Trigrams ####

## Orthography uniqueness point (pu_ortho)

form4$ortho <- as.character(form4$ortho)

form4 <- form4[order(form4$ortho),]

form4$pu_ortho <- 0

head(form4)

for(i in 1:nrow(form4)){

before = 0

after = 0

for(k in 1:form4$nb_letters[i]){

if(match(substr(form4$ortho[i], 1, k), substr(form4$ortho[i-1], 1, k), nomatch = 0) == 1){

before = k

}

if(match(substr(form4$ortho[i], 1, k), substr(form4$ortho[i+1], 1, k), nomatch = 0) == 1){

after = k

}

}

form4$pu_ortho[i] = max(before, after)

}

head(form4)

## Coltheart's N (ortho_neigh)

form4$ortho_neigh <- coltheart.N(form4$ortho, form4$ortho, distance = 1, method = "hamming", parallel = FALSE)

head(form4)

## Orthographical Levenshtein Distance (old20)

form4$old20 <- old20(form4$ortho, form4$ortho, method = "levenshtein", parallel = FALSE)

head(form4)

## Reverse ortography (rev_ortho, rev_cvcv_ortho, rev_bigrams, rev_trigrams)

form4$rev_ortho <- strrev(ortho)

form4$rev_cvcv_ortho <- strrev(cvcv_ortho)

form4$rev_bigrams <- strrev(bigrams)

form4$rev_trigrams <- strrev(trigrams)

## Random value (random)

form4$random <- runif(nrow(form4), 0, 1)

head(form4)

## Order columns

form4 <- form4[,c(,1,2,…)]

head(form4)

## Write table

write.table(form4, file = "C:/…/lexporbr_alfa.txt", sep = "\t")
