# Supplementary material for: The Brazilian Portuguese Lexicon: An Instrument for Psycholinguistic Research
Source: PLoS One. 2015 Dec 2;10(12):e0144016. doi: 10.1371/journal.pone.0144016 (PMC4668042; doi:10.1371/journal.pone.0144016)
Supplement: S3 Text — Algorithm in HTML, CSS, Java, MySQL, and PHP programming languages of the Brazilian Portuguese Lexicon main page (index_en.php). (DOCX) [file pone.0144016.s003.docx]

The Brazilian Portuguese Lexicon: An Instrument for Psycholinguistic Research

Gustavo L. Estivalet^1,2*^ and Fanny Meunier^1,2^

^1^CNRS UMR5304, Laboratoire sur le Langage, le Cerveau et la Cognition, Institut de Sciences Cognitives, Bron, France

^2^Université Claude Bernard Lyon 1, Université de Lyon, Lyon, France

***Corresponding author:** Gustavo Lopez Estivalet, Laboratoire sur le Langage, le Cerveau et la Cognition, Institut de Sciences Cognitives, Bron, France. Phone: +33 651 231 584. E-mail: [gustavo.estivalet@isc.cnrs.fr](mailto:gustavo.estivalet@isc.cnrs.fr) (GLE).

# Supporting Information

**S3 Text. Brazilian Portuguese Lexicon main page algorithm.** Complete algorithm in HTML, CSS, Java, MySQL, and PHP programming languages of the Brazilian Portuguese Lexicon main page (index_en.php).

<?php

//Master query

$query = "SELECT * FROM lexico WHERE ";

//Empty GET

if (empty($_GET))

{

$_GET['criterionS_order'] = "ortografia";

$_GET['criterionC_order'] = "ortografia";

$_GET['criterion1'] = "ortografia";

$_GET['textarea'] = "palavras \r\n em \r\n linhas";

$_POST['nbrow'] = 50;

$query .= "ortografia LIKE '' ";

}

//Simple research

if (!empty($_GET['submit_simple']))

{

$i = 0;

$textarea = $_GET['textarea'];

$criterionS_order = $_GET['criterionS_order'];

$senseS_order = $_GET['senseS_order'];

//Explode textarea

$words = explode("\r\n", $textarea);

foreach($words as $each)

{

$i++;

if ($i == 1)

$query .= "ortografia LIKE ('$each') ";

else

$query .= "OR ortografia LIKE ('$each') ";

}

//Order query

$order = "ORDER BY $criterionS_order $senseS_order ";

}

//Complex research

if (!empty($_GET['submit_complex']))

{

//Define variables

$criterion1 = $_GET['criterion1'];

$criterion2 = $_GET['criterion2'];

$criterion3 = $_GET['criterion3'];

$criterion4 = $_GET['criterion4'];

$equal1 = $_GET['equal1'];

$equal2 = $_GET['equal2'];

$equal3 = $_GET['equal3'];

$equal4 = $_GET['equal4'];

$field1 = $_GET['field1'];

$field2 = $_GET['field2'];

$field3 = $_GET['field3'];

$field4 = $_GET['field4'];

$criterionC_order = $_GET['criterionC_order'];

$senseC_order = $_GET['senseC_order'];

//Check field1

if (!empty($_GET['field1']))

{

//Recognize special characters < and >

$smaller1 = stristr($field1,'<');

$bigger1 = stristr($field1,'>');

//If there is no special characters

if (empty($smaller1 || $bigger1))

$query .= "$criterion1 $equal1 LIKE ('$field1') ";

// If there is <

if (!empty($smaller1))

{

$smaller1 = substr($smaller1,1);

$query .= "$criterion1 $equal1 < ('$smaller1') ";

}

//If there is < and >

if (!empty($smaller1 && $bigger1))

$query .= "AND ";

//If there is >

if (!empty($bigger1))

{

$bigger1 = substr($bigger1,1);

$query .= "$criterion1 $equal1 > ('$bigger1') ";

}

}

//Check field2

if (!empty($_GET['field2']))

{

//If there is in field1

if (!empty($field1))

$query .= "AND ";

//Recognize special characters < and >

$smaller2 = stristr($field2,'<');

$bigger2 = stristr($field2,'>');

//If there is no special characters

if (empty($smaller2 || $bigger2))

$query .= "$criterion2 $equal2 LIKE ('$field2') ";

// If there is <

if (!empty($smaller2))

{

$smaller2 = substr($smaller2,1);

$query .= "$criterion2 $equal2 < ('$smaller2') ";

}

//If there is < and >

if (!empty($smaller2 && $bigger2))

$query .= "AND ";

//If there is >

if (!empty($bigger2))

{

$bigger2 = substr($bigger2,1);

$query .= "$criterion2 $equal2 > ('$bigger2') ";

}

}

//Check field3

if (!empty($_GET['field3']))

{

//If there is in field1 or field2

if (!empty($field1 || $field2))

$query .= "AND ";

//Recognize special characters < and >

$smaller3 = stristr($field3,'<');

$bigger3 = stristr($field3,'>');

//If there is no special characters

if (empty($smaller3 || $bigger3))

$query .= "$criterion3 $equal3 LIKE ('$field3') ";

// If there is <

if (!empty($smaller3))

{

$smaller3 = substr($smaller3,1);

$query .= "$criterion3 $equal3 < ('$smaller3') ";

}

//If there is < and >

if (!empty($smaller3 && $bigger3))

$query .= "AND ";

//If there is >

if (!empty($bigger3))

{

$bigger3 = substr($bigger3,1);

$query .= "$criterion3 $equal3 > ('$bigger3') ";

}

}

//Check field4

if (!empty($_GET['field4']))

{

//If there is in field1 or field2 or field3

if (!empty($field1 || $field2 || $field3))

$query .= "AND ";

//Recognize special characters < and >

$smaller4 = stristr($field4,'<');

$bigger4 = stristr($field4,'>');

//If there is no special characters

if (empty($smaller4 || $bigger4))

$query .= "$criterion4 $equal4 LIKE ('$field4') ";

// If there is <

if (!empty($smaller4))

{

$smaller4 = substr($smaller4,1);

$query .= "$criterion4 $equal4 < ('$smaller4') ";

}

//If there is < and >

if (!empty($smaller4 && $bigger4))

$query .= "AND ";

//If there is >

if (!empty($bigger4))

{

$bigger4 = substr($bigger4,1);

$query .= "$criterion4 $equal4 > ('$bigger4'); ";

}

}

//Order query

$order = "ORDER BY $criterionC_order $senseC_order ";

}

//Create a connection between PHP and MysSQL database

$con = mysqli_connect("server","user","password","database");

mysqli_set_charset($con, 'utf8');

//Check connection

if (mysqli_connect_error())

echo "WARNING: Fail in the connection with MySQL: " . mysqli_connect_error();

//Select the data from criteria

$result = mysqli_query($con, $query);

$totalrow = mysqli_num_rows($result);

$query_export = $query;

//Page displayed

if (!empty($_POST['previous']) || !empty($_POST['next']))

{

$limit = $_POST['nbrow'];

$startrow = $_POST['page'] * $limit;

}

//Define first limits

else

{

$limit = 50;

$_POST['page'] = 0;

$startrow = 0;

}

$page = $_POST['page'] + 1;

$totalpage = ceil($totalrow / $limit);

//Limit min and max of pages and results displayed

if ($startrow < 0)

{

$page = 1;

$startrow = 0;

}

if ($startrow > $totalrow)

{

$page = $totalpage;

$startrow = $page * $limit - $limit;

}

//Order, limit and page displayed

$query .= "$order LIMIT $startrow, $limit ";

$result_page = mysqli_query($con, $query);

$disprow = mysqli_num_rows($result_page);

$endrow = $startrow + $disprow;

//Display statistics

$query_stat = str_replace("*","AVG(freq_orto), AVG(log10_freq_orto), AVG(zipf_escala), AVG(nb_letras), AVG(viz_orto), AVG(old20), MIN(freq_orto), MIN(log10_freq_orto), MIN(zipf_escala), MIN(nb_letras), MIN(viz_orto), MIN(old20), MAX(freq_orto), MAX(log10_freq_orto), MAX(zipf_escala), MAX(nb_letras), MAX(viz_orto), MAX(old20)",$query_export);

$stat = mysqli_query($con, $query_stat);

?>

<!DOCTYPE html>

<html lang="en">

<head>

<meta charset="utf-8">

<style type="text/css">

body, html {margin: 0; padding: 0}

#content {width: 100%; height: 100%}

#head {background: #66CC99; padding: 1%}

#sidebar {width: 12%; height: 300px; float: left; background: #FFFF77; padding: 1%}

#simple {width: 28%; height: 300px; float: left; background: #00DDFF; padding: 1%}

#complex {width: 34%; height: 300px; float: left; background: #00CCFF; padding: 1%}

#tips {width: 18%; height: 300px; float: left; background: #00BBFF; padding: 1%}

#result1 {width: 42%; height: 160px; float: left; background: #CCCCCC; padding: 1%}

#result2 {width: 54%; height: 160px; float: left; background: #DDDDDD; ; padding: 1%; font-size: 10pt}

#result3 {float: left; background: #EEEEEE; ; padding: 1%; font-size: 10pt}

#foot {width: 98%; float: left; background: #66CC99; padding: 1%}

</style>

<title>LexPorBR - Lexicon</title>

</head>

<body bgcolor="#FFFF77">

<div id="content">

<div id="head">

<h1>Brazilian Portuguese Lexicon - LexPorBR</h1>

</div>

<div id="sidebar">

<a href="index.php">Português</a>

<div id="google_translate_element"></div>

<script type="text/javascript">

function googleTranslateElementInit(){

new google.translate.TranslateElement({pageLanguage: 'en', layout: google.translate.TranslateElement.InlineLayout.SIMPLE, autoDisplay: false}, 'google_translate_element');

}

</script>

<script type="text/javascript" src="//translate.google.com/translate_a/element.js?cb=googleTranslateElementInit"></script>

<h3>Links</h3>

<a href="index_en.php">Lexicon</a>

</br><a href="pseudowords_en.php">Pseudowords</a>

</br><a href="downloads_en.php">Downloads</a>

</br><a href="tools_en.php">Tools</a>

</br><a href="updates_en.php">Updates</a>

</br><a href="credits_en.php">Credits</a>

</br><a href="stat_ling_en.php">Statistical Linguistics</a>

</br><a target="_blank" href="http://www.linguateca.pt">Linguateca</a>

</br><a target="_blank" href="http://www.nilc.icmc.usp.br/nilc/index.php">NILC</a>

</br>

</br><a target="_blank" href="/downloads/manual_lexporbr_alpha2_en.pdf">Manual: LexPorBR</a>

</div>

<div id="simple">

<h3>Simple search</h3>

<form method="GET">

<textarea name="textarea" id="textarea" cols="25" rows="6">palavras

em

linhas

</textarea>

<script type="text/javascript">

document.getElementById('textarea').value = "<?php echo $_GET['textarea'];?>";

</script>

</br>

Order by:</br>

<select name="criterionS_order" id="criterionS_order">

<option value="ortografia">orthography</option>

<option value="cat_gram">gram_cat</option>

<option value="inf_gram">gram_inf</option>

<option value="freq_orto">ortho_freq</option>

<option value="freq_orto/M">ortho_freq/M</option>

<option value="log10_freq_orto">log10_ortho_freq</option>

<option value="zipf_escala">zipf_scale</option>

<option value="nb_letras">nb_letters</option>

<option value="nb_homogr">nb_homogr</option>

<option value="homografas">homographs</option>

<option value="pu_orto">pu_ortho</option>

<option value="viz_orto">ortho_neigh</option>

<option value="old20">old20</option>

<option value="cvcv_orto">cvcv_ortho</option>

<option value="bigramas">bigrams</option>

<option value="trigramas">trigrams</option>

<option value="inv_orto">rev_ortho</option>

<option value="inv_cvcv_orto">rev_cvcv_ortho</option>

<option value="inv_bigra">rev_bigrams</option>

<option value="inv_trigra">rev_trigrams</option>

<option value="aleatorio">random</option>

<option value="id">id</option>

</select>

<script type="text/javascript">

document.getElementById('criterionS_order').value = "<?php echo $_GET['criterionS_order'];?>";

</script>

<select name="senseS_order" id="senseS_order">

<option value="">ascendant</option>

<option value="DESC">descendant</option>

</select>

<script type="text/javascript">

document.getElementById('senseS_order').value = "<?php echo $_GET['senseS_order'];?>";

</script>

<input name="submit_simple" type="submit" value="Search">

<button type="reset">Clear</button>

</form>

</div>

<div id="complex">

<h3>Complex search</h3>

<form method="GET">

1 <select name="criterion1" id="criterion1">

<option value="ortografia">orthography</option>

<option value="cat_gram">gram_cat</option>

<option value="inf_gram">gram_inf</option>

<option value="freq_orto">ortho_freq</option>

<option value="freq_orto/M">ortho_freq/M</option>

<option value="log10_freq_orto">log10_ortho_freq</option>

<option value="zipf_escala">zipf_scale</option>

<option value="nb_letras">nb_letters</option>

<option value="nb_homogr">nb_homogr</option>

<option value="homografas">homographs</option>

<option value="pu_orto">pu_ortho</option>

<option value="viz_orto">ortho_neigh</option>

<option value="old20">old20</option>

<option value="cvcv_orto">cvcv_ortho</option>

<option value="bigramas">bigrams</option>

<option value="trigramas">trigrams</option>

<option value="inv_orto">rev_ortho</option>

<option value="inv_cvcv_orto">rev_cvcv_ortho</option>

<option value="inv_bigra">rev_bigrams</option>

<option value="inv_trigra">rev_trigrams</option>

<option value="aleatorio">random</option>

<option value="id">id</option>

</select>

<script type="text/javascript">

document.getElementById('criterion1').value = "<?php echo $_GET['criterion1'];?>";

</script>

<select name="equal1" id="equal1">

<option value="">yes</option>

<option value="NOT">not</option>

</select>

<script type="text/javascript">

document.getElementById('equal1').value = "<?php echo $_GET['equal1'];?>";

</script>

<input name="field1" id="field1" type="text">

<script type="text/javascript">

document.getElementById('field1').value = "<?php echo $_GET['field1'];?>";

</script>

</br>

2 <select name="criterion2" id="criterion2">

<option value="ortografia">orthography</option>

<option value="cat_gram">gram_cat</option>

<option value="inf_gram">gram_inf</option>

<option value="freq_orto">ortho_freq</option>

<option value="freq_orto/M">ortho_freq/M</option>

<option value="log10_freq_orto">log10_ortho_freq</option>

<option value="zipf_escala">zipf_scale</option>

<option value="nb_letras">nb_letters</option>

<option value="nb_homogr">nb_homogr</option>

<option value="homografas">homographs</option>

<option value="pu_orto">pu_ortho</option>

<option value="viz_orto">ortho_neigh</option>

<option value="old20">old20</option>

<option value="cvcv_orto">cvcv_ortho</option>

<option value="bigramas">bigrams</option>

<option value="trigramas">trigrams</option>

<option value="inv_orto">rev_ortho</option>

<option value="inv_cvcv_orto">rev_cvcv_ortho</option>

<option value="inv_bigra">rev_bigrams</option>

<option value="inv_trigra">rev_trigrams</option>

<option value="aleatorio">random</option>

<option value="id">id</option>

</select>

<script type="text/javascript">

document.getElementById('criterion2').value = "<?php echo $_GET['criterion2'];?>";

</script>

<select name="equal2" id="equal2">

<option value="">yes</option>

<option value="NOT">not</option>

</select>

<script type="text/javascript">

document.getElementById('equal2').value = "<?php echo $_GET['equal2'];?>";

</script>

<input name="field2" id="field2" type="text">

<script type="text/javascript">

document.getElementById('field2').value = "<?php echo $_GET['field2'];?>";

</script>

</br>

3 <select name="criterion3" id="criterion3">

<option value="ortografia">orthography</option>

<option value="cat_gram">gram_cat</option>

<option value="inf_gram">gram_inf</option>

<option value="freq_orto">ortho_freq</option>

<option value="freq_orto/M">ortho_freq/M</option>

<option value="log10_freq_orto">log10_ortho_freq</option>

<option value="zipf_escala">zipf_scale</option>

<option value="nb_letras">nb_letters</option>

<option value="nb_homogr">nb_homogr</option>

<option value="homografas">homographs</option>

<option value="pu_orto">pu_ortho</option>

<option value="viz_orto">ortho_neigh</option>

<option value="old20">old20</option>

<option value="cvcv_orto">cvcv_ortho</option>

<option value="bigramas">bigrams</option>

<option value="trigramas">trigrams</option>

<option value="inv_orto">rev_ortho</option>

<option value="inv_cvcv_orto">rev_cvcv_ortho</option>

<option value="inv_bigra">rev_bigrams</option>

<option value="inv_trigra">rev_trigrams</option>

<option value="aleatorio">random</option>

<option value="id">id</option>

</select>

<script type="text/javascript">

document.getElementById('criterion3').value = "<?php echo $_GET['criterion3'];?>";

</script>

<select name="equal3" id="equal3">

<option value="">yes</option>

<option value="NOT">not</option>

</select>

<script type="text/javascript">

document.getElementById('equal3').value = "<?php echo $_GET['equal3'];?>";

</script>

<input name="field3" id="field3" type="text">

<script type="text/javascript">

document.getElementById('field3').value = "<?php echo $_GET['field3'];?>";

</script>

</br>

4 <select name="criterion4" id="criterion4">

<option value="ortografia">orthography</option>

<option value="cat_gram">gram_cat</option>

<option value="inf_gram">gram_inf</option>

<option value="freq_orto">ortho_freq</option>

<option value="freq_orto/M">ortho_freq/M</option>

<option value="log10_freq_orto">log10_ortho_freq</option>

<option value="zipf_escala">zipf_scale</option>

<option value="nb_letras">nb_letters</option>

<option value="nb_homogr">nb_homogr</option>

<option value="homografas">homographs</option>

<option value="pu_orto">pu_ortho</option>

<option value="viz_orto">ortho_neigh</option>

<option value="old20">old20</option>

<option value="cvcv_orto">cvcv_ortho</option>

<option value="bigramas">bigrams</option>

<option value="trigramas">trigrams</option>

<option value="inv_orto">rev_ortho</option>

<option value="inv_cvcv_orto">rev_cvcv_ortho</option>

<option value="inv_bigra">rev_bigrams</option>

<option value="inv_trigra">rev_trigrams</option>

<option value="aleatorio">random</option>

<option value="id">id</option>

</select>

<script type="text/javascript">

document.getElementById('criterion4').value = "<?php echo $_GET['criterion4'];?>";

</script>

<select name="equal4" id="equal4">

<option value="">yes</option>

<option value="NOT">not</option>

</select>

<script type="text/javascript">

document.getElementById('equal4').value = "<?php echo $_GET['equal4'];?>";

</script>

<input name="field4" id="field4" type="text">

<script type="text/javascript">

document.getElementById('field4').value = "<?php echo $_GET['field4'];?>";

</script>

</br>

Order by:</br>

<select name="criterionC_order" id="criterionC_order">

<option value="ortografia">orthography</option>

<option value="cat_gram">gram_cat</option>

<option value="inf_gram">gram_inf</option>

<option value="freq_orto">ortho_freq</option>

<option value="freq_orto/M">ortho_freq/M</option>

<option value="log10_freq_orto">log10_ortho_freq</option>

<option value="zipf_escala">zipf_scale</option>

<option value="nb_letras">nb_letters</option>

<option value="nb_homogr">nb_homogr</option>

<option value="homografas">homographs</option>

<option value="pu_orto">pu_ortho</option>

<option value="viz_orto">ortho_neigh</option>

<option value="old20">old20</option>

<option value="cvcv_orto">cvcv_ortho</option>

<option value="bigramas">bigrams</option>

<option value="trigramas">trigrams</option>

<option value="inv_orto">rev_ortho</option>

<option value="inv_cvcv_orto">rev_cvcv_ortho</option>

<option value="inv_bigra">rev_bigrams</option>

<option value="inv_trigra">rev_trigrams</option>

<option value="aleatorio">random</option>

<option value="id">id</option>

</select>

<script type="text/javascript">

document.getElementById('criterionC_order').value = "<?php echo $_GET['criterionC_order'];?>";

</script>

<select name="senseC_order" id="senseC_order">

<option value="">ascendant</option>

<option value="DESC">descendant</option>

</select>

<script type="text/javascript">

document.getElementById('senseC_order').value = "<?php echo $_GET['senseC_order'];?>";

</script>

<input name="submit_complex" type="submit" value="Search" autofocus>

<button type="reset">Clear</button>

<button type="submit" formmethod="POST" formaction="index_en2.php">+ Fields</button>

</form>

</div>

<div id="tips">

<h3>Use</h3>

_ : to substitute one or more letters

</br>% : to substitute a chain of letters

</br>< : smaller than

</br>> : bigger than

<h3>Grammatical Categories</h3>

adj, adv, gram, nom, num, ver

</div>

<div id="result1">

<h3>Results</h3>

<form method="POST">

<select name="nbrow" id="nbrow">

<option value=50>50</option>

<option value=100>100</option>

<option value=200>200</option>

<option value=500>500</option>

</select>

<script type="text/javascript">

document.getElementById('nbrow').value = "<?php echo $_POST['nbrow'];?>";

</script>

<input name="previous" id="previous" type="submit" value="Previous">

<input name="next" id="next" type="submit" value="Next">

<input name="page" id="page" type="hidden">

<script type="text/javascript">

var count = "<?php echo $_POST['page'];?>";

var next = document.getElementById("next");

var previous = document.getElementById("previous");

next.onclick = function(){

count++;

document.getElementById('page').value = count;

}

previous.onclick = function(){

count--;

document.getElementById('page').value = count;

}

</script>

<button name="export" type="submit" formaction="export_words.php" value="<?php echo $query_export;?>">Export .csv</button>

</form>

<?php

//Display page and words

echo "<br/>Page $page of $totalpage";

echo "<br/>$startrow - $endrow words from a total of $totalrow word found";

?>

</div>

<div id="result2">

<h2>Statistics</h2>

<?php

//Display statistics header

echo "<table border='1'>

<tr>

<th>category</th>

<th>ortho_freq</th>

<th>log10_ortho_freq</th>

<th>zipf_scale</th>

<th>nb_letters</th>

<th>ortho_neigh</th>

<th>old20</th>

</tr>";

//Fetch and display statistics

while($row = mysqli_fetch_array($stat))

{

echo "<tr>";

echo "<td>Mean</td>";

echo "<td>" . $row['AVG(freq_orto)'] . "</td>";

echo "<td>" . $row['AVG(log10_freq_orto)'] . "</td>";

echo "<td>" . $row['AVG(zipf_escala)'] . "</td>";

echo "<td>" . $row['AVG(nb_letras)'] . "</td>";

echo "<td>" . $row['AVG(viz_orto)'] . "</td>";

echo "<td>" . $row['AVG(old20)'] . "</td>";

echo "</tr><tr>";

echo "<td>Min</td>";

echo "<td>" . $row['MIN(freq_orto)'] . "</td>";

echo "<td>" . $row['MIN(log10_freq_orto)'] . "</td>";

echo "<td>" . $row['MIN(zipf_escala)'] . "</td>";

echo "<td>" . $row['MIN(nb_letras)'] . "</td>";

echo "<td>" . $row['MIN(viz_orto)'] . "</td>";

echo "<td>" . $row['MIN(old20)'] . "</td>";

echo "</tr><tr>";

echo "<td>Max</td>";

echo "<td>" . $row['MAX(freq_orto)'] . "</td>";

echo "<td>" . $row['MAX(log10_freq_orto)'] . "</td>";

echo "<td>" . $row['MAX(zipf_escala)'] . "</td>";

echo "<td>" . $row['MAX(nb_letras)'] . "</td>";

echo "<td>" . $row['MAX(viz_orto)'] . "</td>";

echo "<td>" . $row['MAX(old20)'] . "</td>";

echo "</tr>";

}

echo "</table><br/>";

?>

</div>

<div id="result3">

<?php

//Display the table result

echo "<br/><table border='1'>

<tr>

<th>orthography</th>

<th>gram_cat</th>

<th>gram_inf</th>

<th>ortho_freq</th>

<th>ortho_freq/M</th>

<th>log10_ortho_freq</th>

<th>zipf_scale</th>

<th>nb_letters</th>

<th>nb_homogr</th>

<th>homographs</th>

<th>pu_ortho</th>

<th>ortho_neigh</th>

<th>old20</th>

<th>cvcv_ortho</th>

<th>bigrams</th>

<th>trigrams</th>

<th>rev_ortho</th>

<th>rev_cvcv_ortho</th>

<th>rev_bigrams</th>

<th>rev_trigrams</th>

<th>random</th>

<th>id</th>

</tr>";

//Fetch and display results

while($row = mysqli_fetch_array($result_page))

{

echo "<tr>";

echo "<td>" . $row['ortografia'] . "</td>";

echo "<td>" . $row['cat_gram'] . "</td>";

echo "<td>" . $row['inf_gram'] . "</td>";

echo "<td>" . $row['freq_orto'] . "</td>";

echo "<td>" . $row['freq_orto/M'] . "</td>";

echo "<td>" . $row['log10_freq_orto'] . "</td>";

echo "<td>" . $row['zipf_escala'] . "</td>";

echo "<td>" . $row['nb_letras'] . "</td>";

echo "<td>" . $row['nb_homogr'] . "</td>";

echo "<td>" . $row['homografas'] . "</td>";

echo "<td>" . $row['pu_orto'] . "</td>";

echo "<td>" . $row['viz_orto'] . "</td>";

echo "<td>" . $row['old20'] . "</td>";

echo "<td>" . $row['cvcv_orto'] . "</td>";

echo "<td>" . $row['bigramas'] . "</td>";

echo "<td>" . $row['trigramas'] . "</td>";

echo "<td>" . $row['inv_orto'] . "</td>";

echo "<td>" . $row['inv_cvcv_orto'] . "</td>";

echo "<td>" . $row['inv_bigra'] . "</td>";

echo "<td>" . $row['inv_trigra'] . "</td>";

echo "<td>" . $row['aleatorio'] . "</td>";

echo "<td>" . $row['id'] . "</td>";

echo "</tr>";

}

echo "</table>";

mysqli_close($con);

?>

</br>

</div>

<div id="foot">

<a target="_blank" href="http://creativecommons.org/licenses/by-nc-sa/4.0/"><img alt="Creative Commons License" style="border-width:0" src="http://i.creativecommons.org/l/by-nc-sa/4.0/88x31.png"/></a></br><a href="http://www.lexicodoportugues.com/">Brazilian Portuguese Lexicon</a> is licensed with a License <a target="_blank" href="http://creativecommons.org/licenses/by-nc-sa/4.0/">Creative Commons - Attribution-NonCommercial-ShareAlike 4.0 International</a>.

</br>Last update: 12/10/2015.

</div>

</div>

</body>

</html>
